# Supplementary material for: A supramolecular gel-elastomer system for soft iontronic adhesives
Source: Nat Commun. 2023 Apr 8;14:1990. doi: 10.1038/s41467-023-37535-4 (PMC10082814; doi:10.1038/s41467-023-37535-4)
Supplement: Supplementary file 1 — Supplementary Information [file 41467_2023_37535_MOESM1_ESM.pdf]

Supplementary Information

A supramolecular gel-elastomer system for soft iontronic adhesives

Dace Gao[1†], Gurunathan Thangavel[1†§], Junwoo Lee[2†§], Jian Lv[1,3], Yi Li[4], Jing-Hao Ciou[1], Jiaqing Xiong[1], Taiho Park[3], and Pooi See Lee[1,3]*

[1]School of Materials Science and Engineering, Nanyang Technological University, 50 Nanyang Avenue, Singapore 639798, Singapore

[2]Department of Chemical Engineering, Pohang University of Science and Technology, Pohang 37673, Republic of Korea

[3]Singapore-HUJ Alliance for Research and Enterprise (SHARE), Smart Grippers for Soft Robotics (SGSR), Campus for Research Excellence and Technological Enterprise (CREATE), Singapore 138602, Singapore

[4]School of Electrical Engineering and Automation, Wuhan University, Wuhan 430072, China

[†]These authors contributed to this work equally

[§]Present address: Advanced Materials Research Center, Technology Innovation Institute (TII), Masdar City, Abu Dhabi, P.O Box 9639, United Arab Emirates (T.G.); Department of Chemical and Environmental Engineering, Yale University, New Haven, CT 06511, USA (J.L.)

*e-mail: pslee@ntu.edu.sg

This PDF file includes

Supplementary Methods

Supplementary Table 1-3

Supplementary Fig. 1-32

Legends for Supplementary Video 1-8

References

Supplementary Methods

Synthesis of P(SPMA$_{0.5}$-r-MMA$_{0.5}$)

P(SPMA$_{0.5}$-r-MMA$_{0.5}$) polyelectrolyte was synthesized by atom transfer radical polymerization (ATRP) to obtain controllable M_n and smaller PDI. P(SPMA$_{0.5}$-r-MMA$_{0.5}$) was also synthesized by free radical polymerization (FRP) for comparison. In ATRP method (Supplementary Fig. 1a), **Monomer 1** (3-sulfopropyl methacrylate potassium salt, SPMA) (4.9 g, 20.0 mmol), **catalyst** (copper(I) bromide, CuBr) (14.3 mg, 0.1 mmol), and **ligand** (*N*,*N*,*N′*,*N″*,*N″*-Pentamethyldiethylenetriamine, PMDETA) (17.3 mg, 0.1 mmol) were firstly dissolved in 12 ml water/DMF cosolvent (40/60 vol%). The mixture consisting of **monomer 2** (methyl methacrylate, MMA) (2.0 g, 20.0 mmol), **initiator** (Ethyl α-bromoisobutyrate, EBiB) (19.5 mg, 0.1 mmol), and 3 ml cosolvent was then incorporated and the solution was stirred at 70 °C for 50 h. After that, the polyelectrolyte was precipitated in DMF (4.9 g, 71% yield, colorless solid). In FRP method (Supplementary Fig. 1b, adopted in our previous work[1]), **Monomer 1** (3-sulfopropyl methacrylate potassium salt, SPMA) (4.9 g, 20.0 mmol), **monomer 2** (methyl methacrylate, MMA) (2.0 g, 20.0 mmol), and **catalyst** (benzoyl peroxide, BPO, Luperox A75) (12.1mg, 0.05 mmol) were dissolved in 7 ml water/dimethylformamide (DMF) cosolvent (50/50 vol%) for polymerization at 70 °C for 12 h. The polyelectrolyte was then precipitated in MeOH (6.8g, 98% yield, colorless solid).

Synthesis of SHPU

Concept of macromolecular design | A classical thermoplastic polyurethane (TPU) is composed of isolated hard domains embedded in a soft matrix, wherein the rubbery matrix gives rise to stretchability while the semicrystalline domains (i.e. rigid fillers) physically crosslink the polymeric network and contribute to high mechanical strength. While introducing kinetically labile crosslinking sites represents a feasible strategy to enable room-temperature self-restoration in many elastomers, such a practice in TPU is likely to impair the crystallizability of hard domains and thus weaken their inherent robustness. We envisioned that through supramolecular phase engineering, UPy motifs carrying quadruple H-bonding sites could be introduced within the soft matrix to decouple from the semicrystalline hard phase and further harness the dynamic chain motion in the soft phase to facilitate self-healing under ambient conditions. To fulfill this concept, SHPU end-functionalized with UPy was synthesized through step-growth polymerization. Specifically, PU macromers comprising polytetramethylene ether glycol (PTMEG)-based soft segments and isophorone-based hard segments were first synthesized, then joined

into a branched network via triethanolamine (TEA, a trifunctional chain extender). UPy units were subsequently incorporated as associating terminal groups. Upon thermal curing, HSs self-assemble into rigid aggregations via inter-urethane H-bonding, while SSs are reversibly associated by self-complementary UPy dimers.

Chemicals | Polytetramethylene ether glycol (PTMEG, Sigma-Aldrich, $M_n \approx 1000$) and 1,4-butenediol (1,4-BuD, Sigma-Aldrich, 99%) were dried in vacuum oven at 100 °C for 1 h before usage. Isophorone diisocyanate (IPDI, Alfa Aesar, mixture of isomers, 98%), hexamethylene diisocyanate (HDI, Sigma-Aldrich, ≥ 99%), guanidine carbonate (GDN, Sigma-Aldrich, 99%), triethanolamine (TEA, Sigma-Aldrich, 98%) ethyl acetoacetate (EAA, Sigma-Aldrich, 99%), and *N,N´*-dimethylacetamide (DMAc, Alfa Aesar, anhydrous 99.8%) were used without further purification. Dibutyltin dilaurate (DBTDL, 95%), pyridine (=CH-, anhydrous, 99.8%), pentane (anhydrous, ≥ 99%), acetone (anhydrous, ≥ 99.5%), chloroform ($CHCl_3$, ≥ 99.5%), diethyl ether (DEE, anhydrous, ≥ 99.7%) and other chemicals were purchased from Sigma-Aldrich and used as received unless otherwise specified.

Synthesis of 2-Amino-4-hydroxy-6-methylpyrimidine or 6-methylisocytosine (MIC) | The scheme of MIC synthesis is presented in Supplementary Fig. 2a. Specifically, a suspension of GDN (0.16 mol) and EAA (0.36 mol) in ethanol (150 ml) was heated under reflux for 12 h, then filtered with a büchner funnel to receive a white paste-like mixture. The mixture was kept at 0 °C for 0.5 h, and subsequently washed with ethanol, deionized (DI) water, and acetone in sequence. The dissolution-precipitation-decantation process was repeated for five times and the final product was dried at 50 °C under vacuum overnight to obtain MIC.

Synthesis of 1-(6-isocyanatohexyl)-3-(6-methyl-4-oxo-1H-pyrimidin-2-yl)urea (UPy-NCO) | The synthesis of UPy-NCO (Supplementary Fig. 2b) was performed following a previously reported procedure.[2] Specifically, MIC (60 mmol), 1,6-hexamethylene diisocyanate (350 mmol) and pyridine (8 mL) were added into a flask and reacted at 100 °C (24 h, N_2 atmosphere) with magnetic stirring and reflux condensation. After that, the flask was cooled down to room temperature, followed by adding 50 mL n-pentane to quench the reaction. The mixture was then kept at 0 °C for 0.5 h to precipitate, and the upper clear solution was decanted. The precipitation was rinsed by acetone then subjected to vacuum evaporation at 50 °C to obtain the UPy-NCO powder.

Synthesis of supramolecular, hierarchically H-bonded polyurethane (SHPU) | The dynamically crosslinked SHPU was synthesized through step-growth polymerization as depicted in Supplementary Fig. 3. PTMEG (29 mmol) was firstly fed into a flask and heated at 100 °C for 1 h to remove residue moisture. IPDI (63.95 mmol) and DBTDL (0.06 wt%) dissolved in DMAc were then added dropwise into the flask and stirred at 85 °C (3 h, N_2 atmosphere) for primary isocyanate reaction **(Step I)**. Afterwards, 1,4-BuD (14.50 mmol) in 10 mL anhydrous DMAc was added to the prepolymer solution and allowed for secondary isocyanate reaction at 75 °C (4 h, N_2 atmosphere) **(Step II)**. After the synthesis of OCN-PU-NCO, TEA (14.50 mmol) and UPy-NCO (4.5 mmol) were introduced and stirred until fully dissolved. Reactions of chain extending and telechelic UPy jointing were carried out at 75 °C (3 h, N_2 atmosphere) to get the SHPU resin **(Step III)**, which could be further drop casted on glass and cured (90 ^{o}C, 12 h) to remove the solvent and achieve a transparent elastomer film.

Supplementary Tables

Supplementary Table 1 | Assignment of characteristic peaks and amide vibration modes in SHPU. v = stretching, δ = bending.

Raman shift (cm^{-1})	Assignment
3330	v(N–H)
2932 & 2860	v_s(C–H), v_a(C–H)
2256	v(N=C=O)
1760-1630	Amide I: v(C=O)[6]
1720	v(C=O) in free urethane and free urea
1695	v(C=O): H-bonded in urethane
1665	v(C=O): H-bonded in UPy isocytosine[7]
1536	Amide II: v(C–N) + δ(N–H) + v(C–C)[6]
1521	v(N–H): H-bonded ureido N-H in UPy urea[7]
1456	δ(C–H): CH_2 scissoring, CH_3 deformation and CH_2 bending
1367	v(C–N) & (C–C–N) deformation
1237	Amide III: v(C–N) and in-plane (N-H) deformation[6]
1100	v(C–O–C) in ether group & v(C–N)
770	Amide IV: N-H out of plane deformation[6]

Supplementary Table 2 | Comparison of various room-temperature self-healable elastomers in terms of their mechanical properties and self-healing efficiency.

	Pristine			Healed				
Healing motif	Elongation at break (%)	UTS (MPa)	Toughness (MJ m^{-3})	Elongation at break (%)	UTS (MPa)	Toughness (MJ m^{-3})	Healing time (h)	Ref.
**H-bond (this work)**	**2000**	**14.4**	**100.8**	**1840**	**10.1**	**72.9**	**24**	/
H-bond	2010	4.8	65.5	2000	4.8	64.0	48	[8]
H-bond	1800	1.7	14.8	1580	1.3	11.1	48	[9]
H-bond	600	3.4	8.0	520	2.9	6.0	3	[10]
H-bond	780	1.9	10.0	710	1.7	8.0	24	[11]
M-L coordination	1070	2.5	29.3	1060	2.4	27.8	24	[12]
M-L coordination	1850	0.2	3.8	1700	0.2	3.4	48	[13]
M-L & H-bond	1200	14.8	87.0	760	8.0	38.1	40	[14]
M-L & H-bond	900	1.8	6.0	770	1.3	4.6	48	[15]
Disulfide	3100	0.8	13.0	2625	0.7	8.5	2	[16]
Disulfide	923	6.8	26.9	920	6.0	20.6	2	[17]
Diselenide	700	2.1	6.6	620	1.6	4.5	24	[18]
van der Waals	560	4.4	12.0	500	4.4	11.0	120	[19]
Catechol	310	3.5	6.8	190	4.2	5.3	24	[20]

Supplementary Table 3 | Payload comparison of the reported electrostatic-adhesive grippers. *A paper coating is applied on the acrylic to avoid dry adhesion (Van der Waals force).

Reference	Gripper's weight (n)	Load (m)	Driving voltage	Payload (m/n)
Ref[21]	1.5 g	82.1 g (metallic can)	3.5 kV	54.7
Ref[22]	1.5 g	16 N (acrylic geometry connected to a force sensor)	3.5 kV	1088.4*
Ref[23]	6.2 g	625 g (glass bottle filled with metal screws)	Ï7 kV	100.8
This work	0.32 g	215 g (metallic cube and weights)	1 kV	671.9

Supplementary Figures

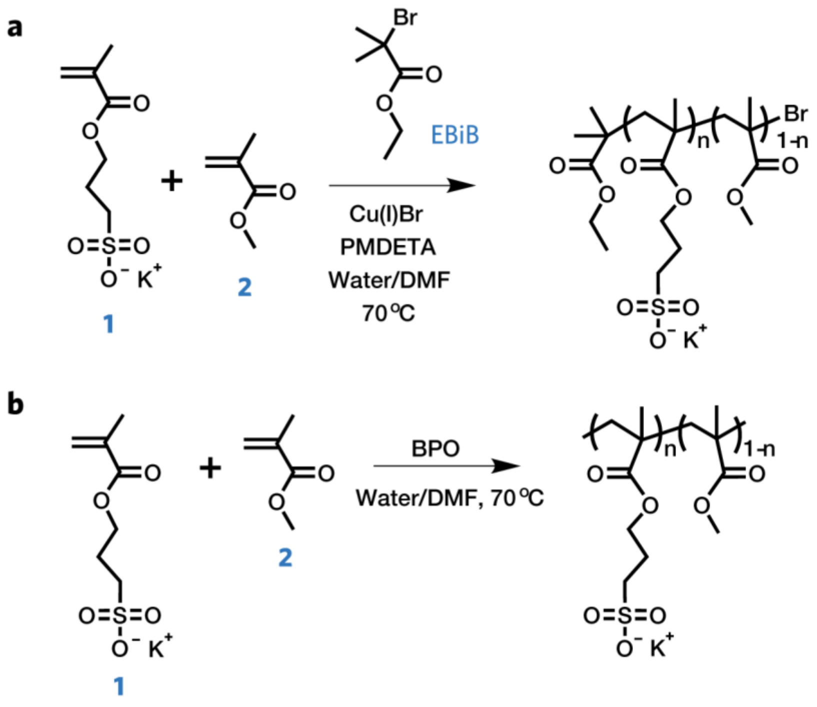

Supplementary Fig. 1 | Synthesis of P(SPMA$_{0.5}$-r-MMA$_{0.5}$). a, ATRP method. **b,** FRP method.

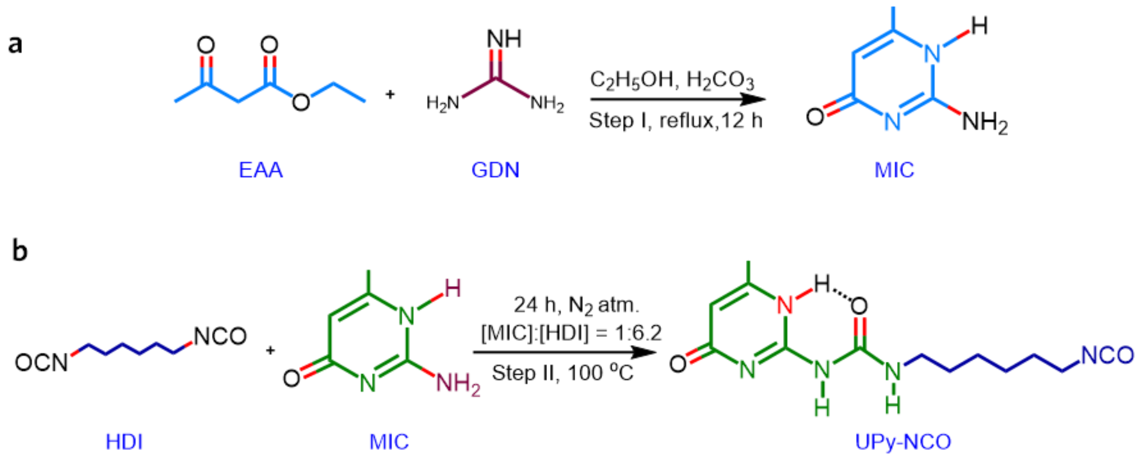

Supplementary Fig. 2 | Synthetic routes for SHPU precursors. a, Synthesis of MIC. **b,** Synthesis of UPy-NCO.

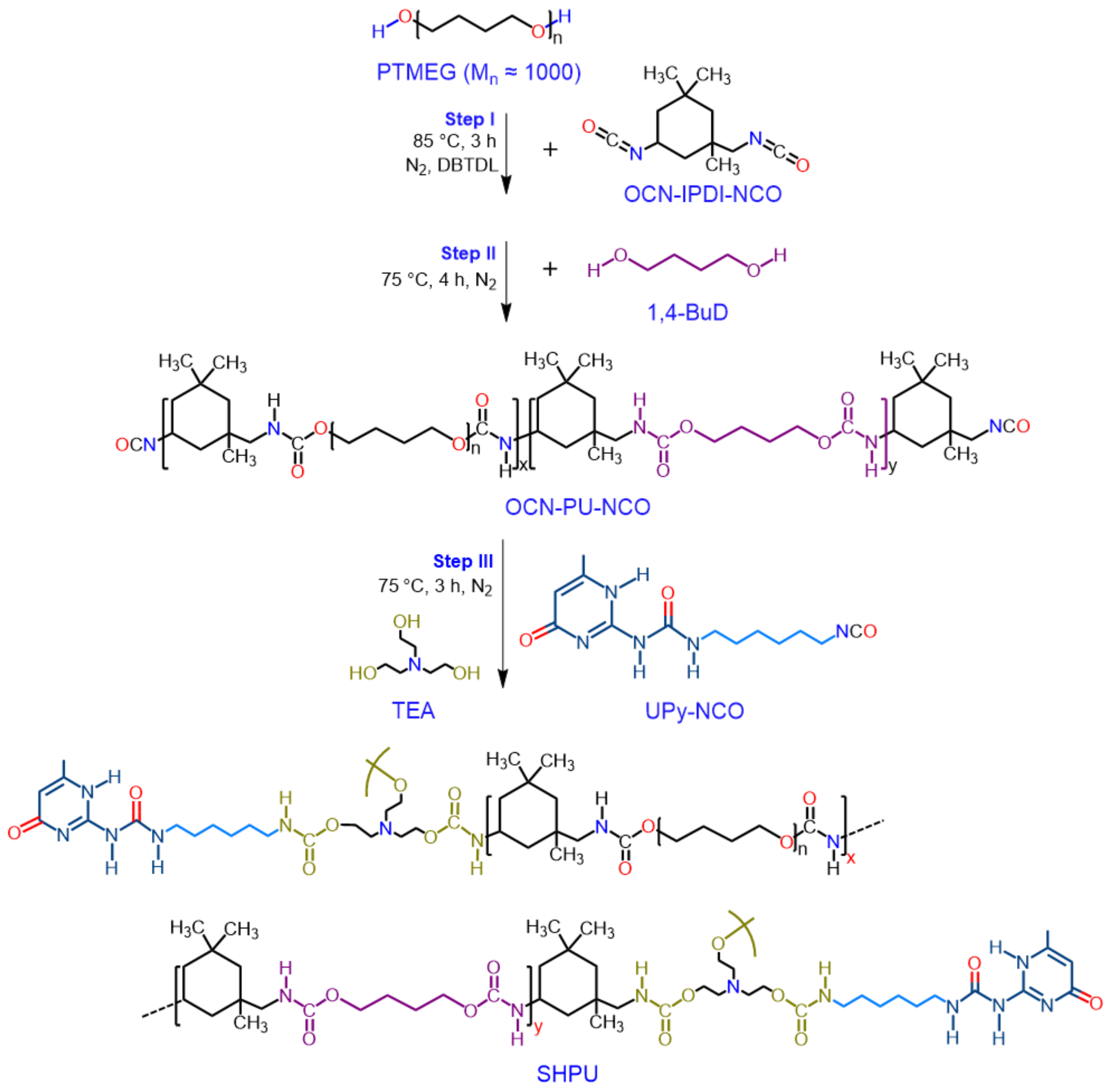

Supplementary Fig. 3 | Synthetic route of SHPU.

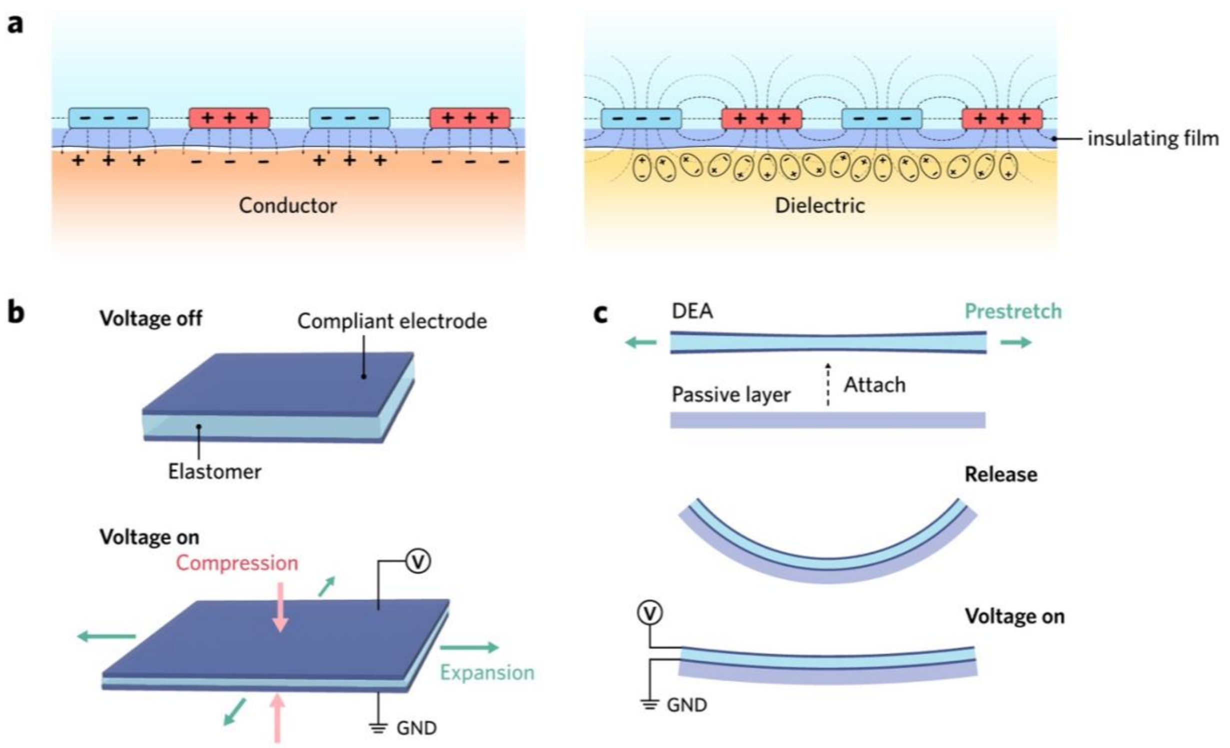

Supplementary Fig. 4 | Mechanisms of electrostatic adhesion and actuation. a-c, Schematic illustrations showing the mechanisms for **(a)** electrostatic adhesion on conductive and dielectric surfaces, **(b)** the mechanism for electrostatic actuation, and **(c)** the formation and bending mechanism of DEMES unimorph.

Mechanisms for electrostatic adhesion:

Electrostatic adhesion is an engineering method that generates astrictive forces between electroadhesive patches and a broad range of substrates. An electrostatic adhesive patch generally consists of a set of interdigitated electrodes and an insulating film. When the electrodes are activated (one subjected to high voltage input, the other grounded), electrostatic attraction between the electrodes and an opposing substrate will generate through electrostatic induction or polarization. For our iontronic adhesives, the interdigitated electrode is ionic gel (OHGel) with ionic conductivity, while the insulating layer is dielectric elastomer (SHPU).

The mechanism of electrostatic adhesion varies depending on the material composition of an opposing substrate. When a powered electroadhesive patch is applied onto a conductive substrate, the strong electric fringe field will induce charge separation in the conductor and consequently form a set of parallel-plate capacitors between the electrodes and the metallic surface (Supplementary Fig. 4a, left). The adhesion pressure (P_{ad}) is given by

$$P_{ad} = \frac{1}{2}\varepsilon_0\varepsilon\left(\frac{V}{d}\right)^2 \tag{1}$$

where ε_0 is vacuum permittivity, ε is the dielectric constant of the insulating film, d is the thickness of the insulating film, or the distance from the electrode plane to the foreign substrate surface. Dielectric materials possess no mobile charge carriers and are thereby polarized in response to an external electric field (Supplementary Fig. 4a, right). The strength of adhesion is positively correlated to the total amount of surface polarization, or dipole moment, of the dielectric substrate.

Mechanisms for electrostatic actuation:

A basic DEA consists of a dielectric elastomer film sandwiched by a pair of compliant electrodes. When a large voltage drop is applied, the elastomer will be compressed in response to the electrostatic attraction generated from the charges on the opposing electrodes (Supplementary Fig. 4b). The attraction is described as Maxwell pressure (P) and is quantified as

$$P = \varepsilon_0\varepsilon\left(\frac{V}{t}\right)^2 \tag{2}$$

where ε_0 is vacuum permittivity, ε is the dielectric constant of the dielectric elastomer, and t is the thickness of the dielectric layer. At relatively small strains (< 20%), the compressive strain in thickness direction is estimated by

$$P = \frac{1}{Y}\varepsilon_0\varepsilon\left(\frac{V}{t}\right)^2 \tag{3}$$

where Y is the Young's modulus of the elastomer. Furthermore, as elastomeric materials have constant volume under uniaxial stress, in-plane (lateral) expansion can be produced in compensation to the contraction in thickness.

Bending in DEMES:

A dielectric elastomer minimum energy structure (DEMES) consists of a DEA whose dielectric film is prestretched and then adhered to a passive layer. Compliant electrodes are applied on both sides of the prestretched film. The passive layer can be either flexible plastics or stretchable elastomers. The tension in the prestretched film causes the overall structure to bend until the bending energy in the passive layer equals to the strain energy in DEA, and the structure reaches its minimum energy equilibrium. Upon high voltage stimulation, the dielectric membrane in DEA expands laterally and cancels its contractile restriction to the passive layer, thus allows the DEMES to flatten (Supplementary Fig. 4c). In our gripping unit (refer to Fig. 4a), SHPU layer (3) is the prestretched dielectric membrane, while SHPU layer (5) and (6) together form the passive layer.

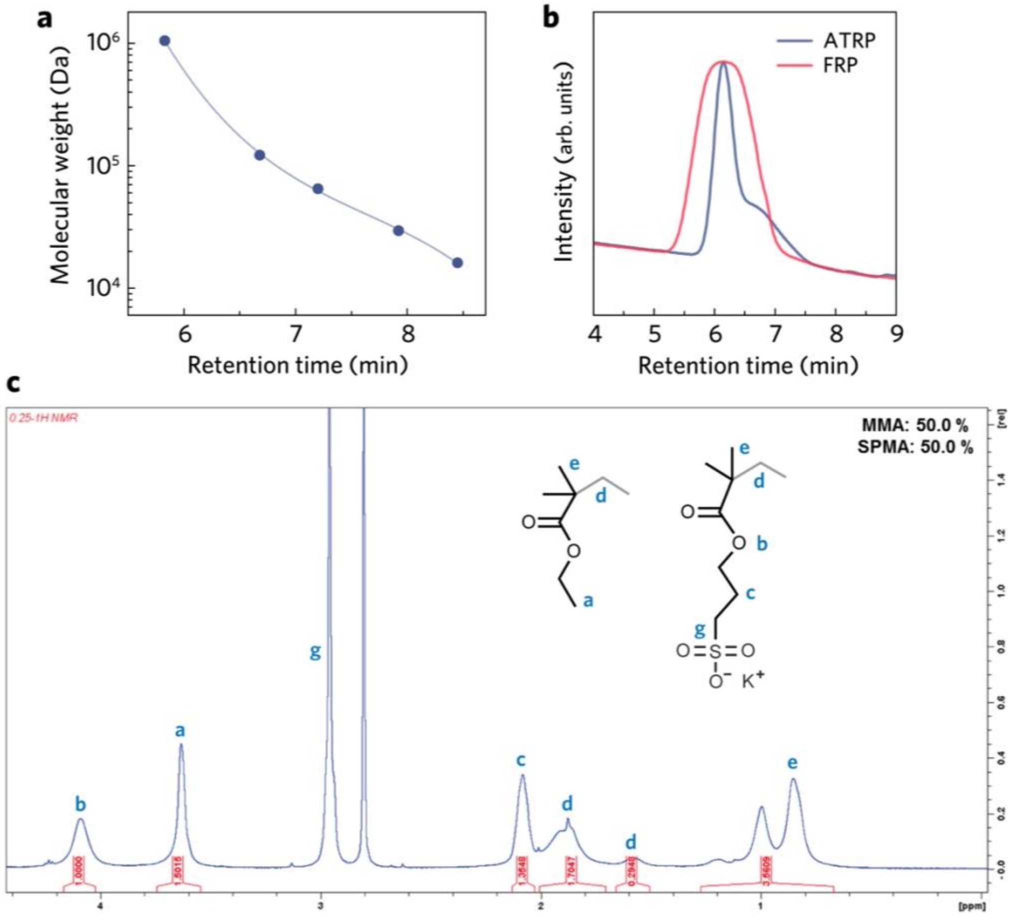

Supplementary Fig. 5 | Characterization of P(SPMA$_{0.5}$-r-MMA$_{0.5}$). a, GPC calibration plot derived from a set of standard chromatograms. **b,** GPC traces indicating the retention times for both ATRP and FRP polyelectrolytes. ATRP-GPC (CB, 40 ℃): M_n = 180.0 K, M_w = 325.8 K, PDI = 1.81. FRP-GPC (CB, 40 ℃): M_n = 247.0 K, M_w = 718.8 K, PDI = 2.91. Polyelectrolyte synthesized via ATRP has lower M_n and PDI due to the reduced propagation rate during chain growth. The narrower distribution in M_n is favored by drop-on-demand (DOD) inkjet printing as the polymer chains can undergo coil-stretch transition during fluid ejection without producing the antagonistic restoring force[24]. **c,** ^{1}H NMR of ATRP-P(SPMA$_{0.5}$-r-MMA$_{0.5}$) (600 MHz, D_2O, δ/ppm): 4.21-4.00 (br, -CH_2), 3.78-3.59 (br, -CH_3), 3.08-2.95 (br, -CH_2), 2.18-2.08 (br, -CH_2), 2.08-1.75 (br, -CH_2), 1.68-1.53 (br, -CH_2), 1.28-0.79 (br, -CH_3).

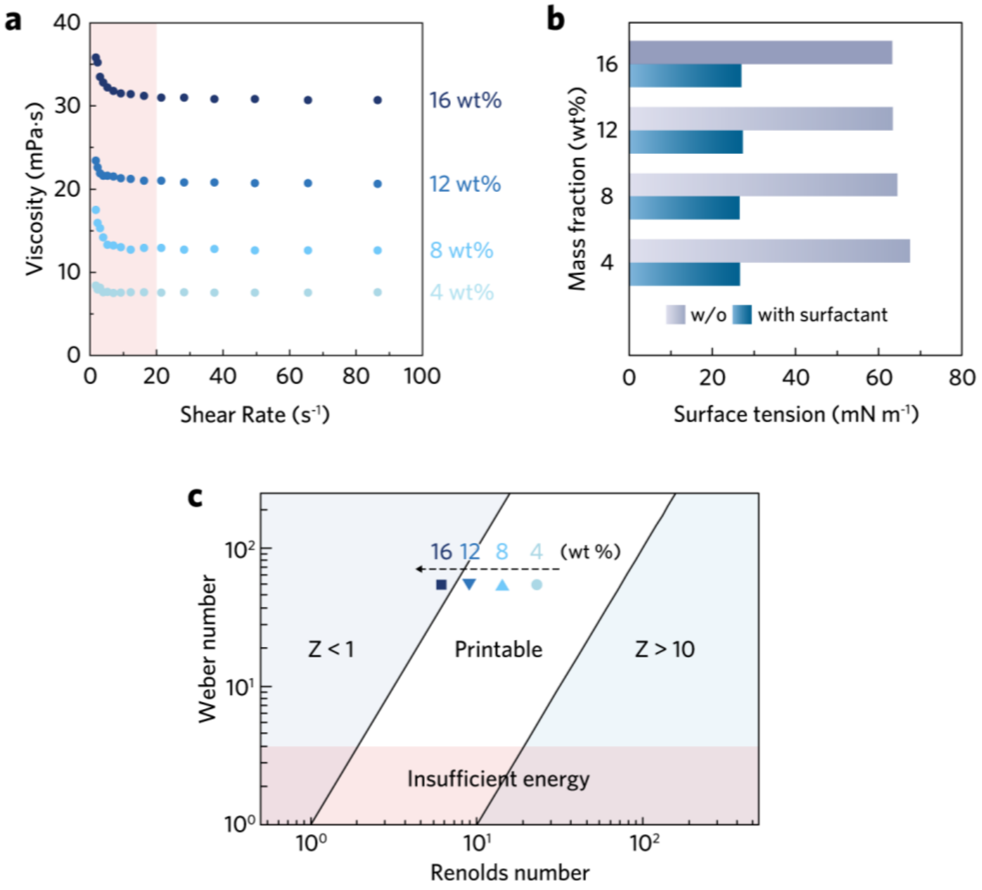

Supplementary Fig. 6 | Inkjet printability of the pre-OHGel inks. a, Apparent viscosity of the inks with varying polyelectrolyte load as a function of shear rate. **b,** Surface tension of the inks with or without the addition of surfactant. **c,** We-Re dimensionless space suggesting the printability of the pre-OHGel inks with varying polyelectrolyte load.

The counterbalance between inertial force, capillary force and the fluid/air interfacial tension controls the dynamics of droplet generation in DOD printer head.[25] These underlying physical effects can be analyzed by several dimensionless groups of physical constants, including Weber (*We*) and Reynold (*Re*) numbers:

$$We = \frac{\rho v^2 a}{\gamma} \qquad Re = \frac{\rho v a}{\eta}$$

where the values are determined by ink density (ρ), viscosity (η), surface tension (γ), jetting velocity (v) and nozzle aperture (a). We investigated the rheological property of the pre-OHGel ink by varying the mass fraction of P(SPMA$_{0.5}$-r-MMA$_{0.5}$) from 4 to 16 wt% while fixing the PE/GY mass ratio at 2:1 in

the inks. The apparent viscosity-shear rate (η - γ) plots in Supplementary Fig. 6a indicate a shear-thinning behavior of the inks at low-shear region (< 20 s^{-1}), whose trend can be interpreted by Ostwald–de Waele power law. At high-shear region (> 20 s^{-1}), viscosity values are stable and exhibit a positive correlation with the load of polyelectrolyte. Besides, the addition of 0.02 wt% polymeric surfactant can regulate the inks' surface tension to ~27 mN m^{-1} (Supplementary Fig. 6b). The parameter $Z = Re/\sqrt{We}$ serves as a figure of merit to evaluate the capability of droplet formation,[25,26] whereby a rational range of $1 < Z < 10$ can be mapped into the *We*-*Re* coordinate space (Supplementary Fig. 6c) to determine if an ink is inkjet printable. Based on these characterizations, we find a printable pre-OHGel ink has an upper limit of ~12 wt% in PVA concentration, above which the viscous dissipation will prevent fluid ejection. As a result, pre-OHGel ink loaded with 8 wt% polyelectrolyte and 4 wt% glycerol was selected for this study given its optimal jetting performance and fast gelating rate at room temperature. For ink-substrate interaction, the surface energy of pristine SHPU couples well with that of OHGel ink and allows for uniform OHGel deposition.

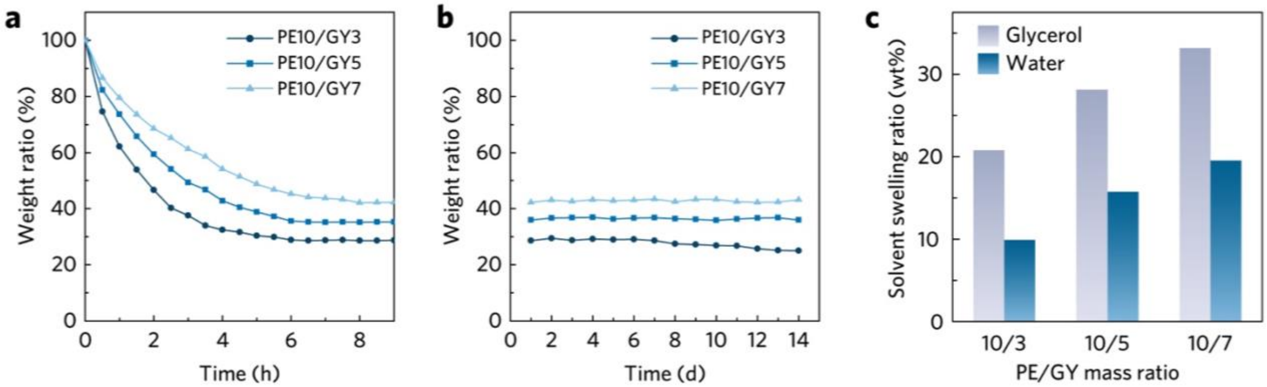

Supplementary Fig. 7 | a, Initial weight loss in OHGels during the partial dehydration and gelation process after inkjet printing. Weight ratios are calculated by dividing measured weight by initial weight. **b,** Net weight change in OHGels recorded up to 14 days (ambient condition, RH ≈ 60%). No noticeable mass change in PE10/GY7 and PE10/GY5 was observed. The slight fluctuation in weight could be due to the fluctuation in relative humidity. PE10/GY3 further lost ~ 3.6 wt% on day 14 due to its lower glycerol content. **c,** Weight fraction of water and glycerol in OHGels (in equilibrium with atmospheric moisture, RH ≈ 60%).

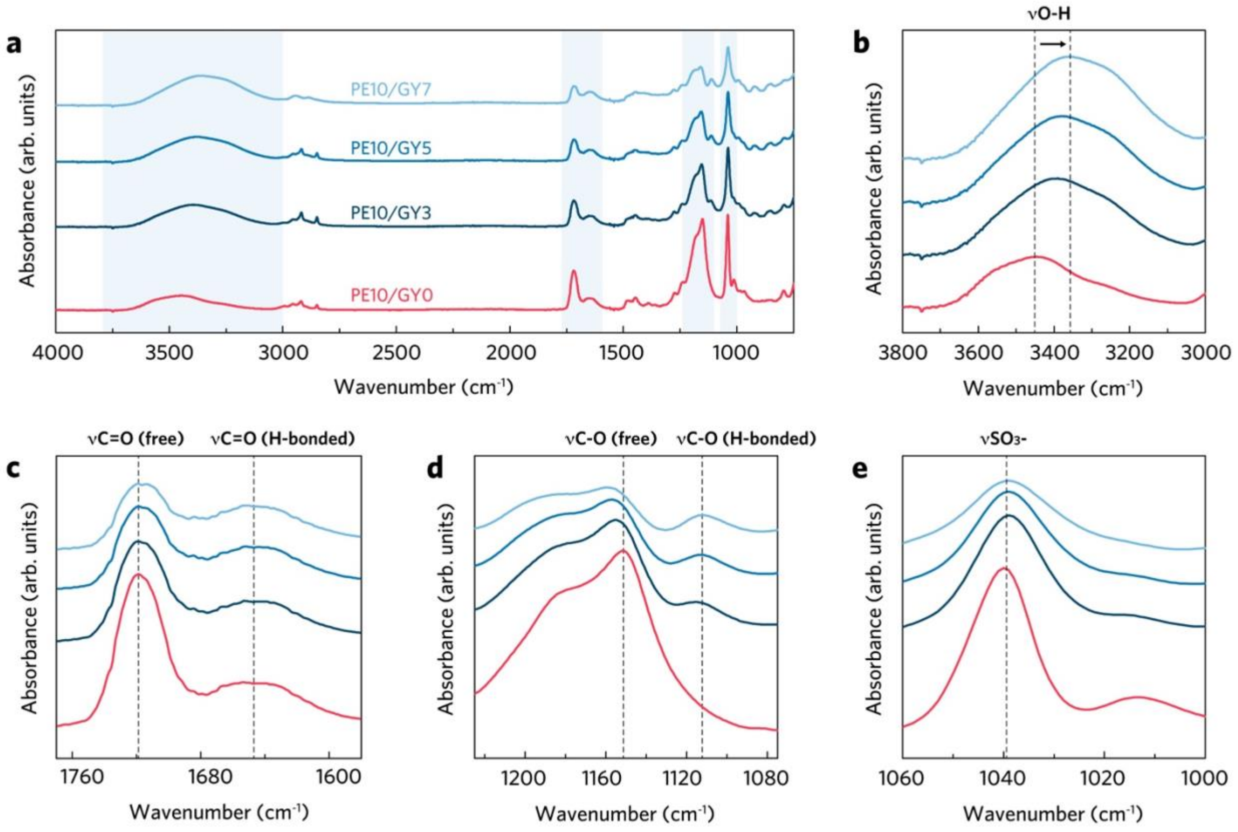

Supplementary Fig. 8 | FTIR characterization revealing the solvent-polyelectrolyte interaction in OHGels. a, ATR-FTIR spectra of pure P($SPMA_{0.5}$-r-$MMA_{0.5}$) (red) and OHGels with different PE/GY mass ratio (blue). **b-e,** Magnified spectral views in the wavenumber range of **(b)** 3800 cm^{-1} to 3000 cm^{-1}, **(c)** 1770 cm^{-1} to 1580 cm^{-1}, **(d)** 1225 cm^{-1} to 1075 cm^{-1}, and **(e)** 1060 cm^{-1} to 1000 cm^{-1}.

The interplay between glycerol and P($SPMA_{0.5}$-r-$MMA_{0.5}$) at molecular level was revealed via ATR-FTIR. Characteristic peaks of P($SPMA_{0.5}$-r-$MMA_{0.5}$) include free C=O stretching in ester group at 1720 cm^{-1} (Supplementary Fig. 8c), free C-O stretching in aliphatic ether at 1150 cm^{-1} (Supplementary Fig. 8d), and the fingerprint peak of $-SO_3^-$ at 1040 cm^{-1} (Supplementary Fig. 8e). The term “free” in this context denotes C=O and C-O groups that are not H-bonded. O-H stretching at 3440 cm^{-1} in the spectrum of “pure” P($SPMA_{0.5}$-r-$MMA_{0.5}$) suggests the existence of bound water due to its hygroscopicity. The plasticizing effect of GY could be unraveled by analyzing the relative strength and infrared characteristics of intermolecular hydrogen bonds. Upon the inclusion of glycerol, the O-H to C-O interaction gives rise to a signature peak of H-bonded C-O band at 1112 cm^{-1} and reduces the relative intensity of free C-O stretching states at 1150 cm^{-1} (Supplementary Fig. 8d). Similarly, the increased

relative strength of H-bonded C=O band (centered at 1645 cm^{-1}) to free C=O suggests that a higher fraction of C=O become H-bonded along the increment of glycerol content (Supplementary Fig. 8c), and the peak shift of O-H stretching towards lower wavenumber also indicates an increased amount of intermolecular hydrogen bonding (Supplementary Fig. 8b).

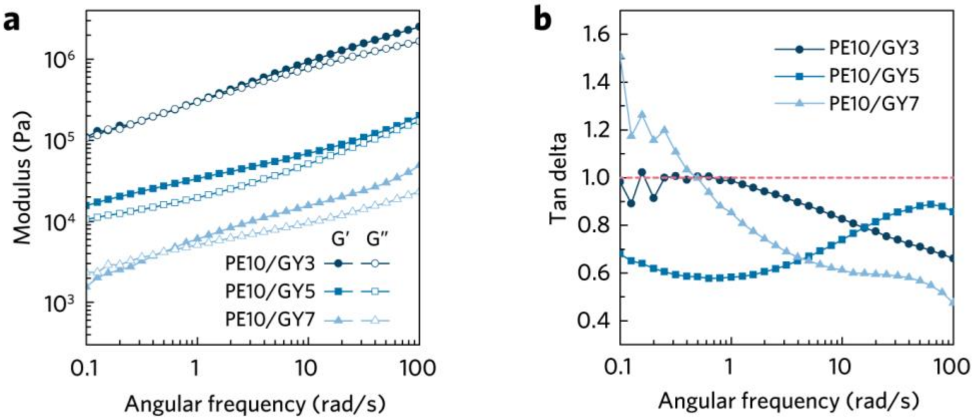

Supplementary Fig. 9 | Rheological characterization of OHGels. a, Shear storage modulus and loss modulus as a function of angular frequency for OHGels of varying PE/GY mass ratio. **b,** Tan δ (G"/G') as a function of angular frequency for OHGels of varying PE/GY mass ratio. Among all the samples, PE10/GY5 exhibits the best elasticity with G' higher than G" (tan $\delta < 1$) throughout the frequency regime. G" of PE10/GY3 and PE10/GY7 surpasses G' in low frequency region (tan $\delta > 1$), as the deformation timescale becomes longer than their characteristic relaxation time and leads to a viscous flowing behavior. As such, modifying the composition of glycerol enables us to design a series of OHGel species with a wide spectrum of mechanical properties.

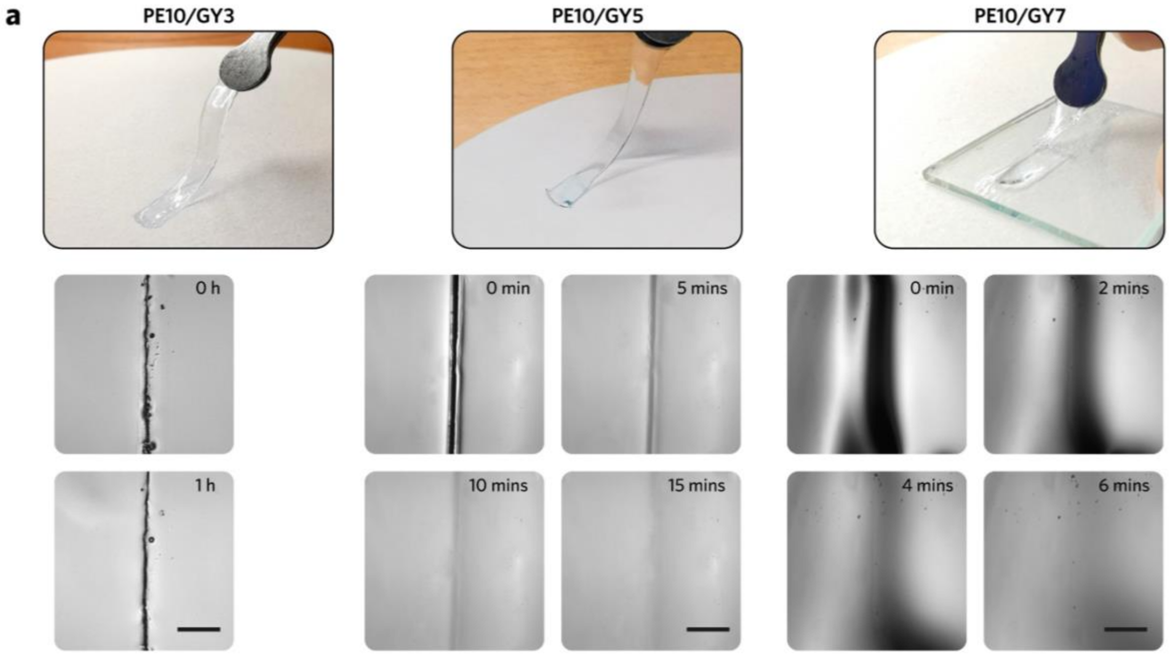

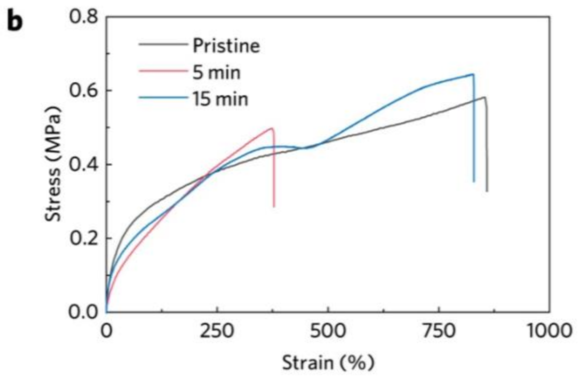

Supplementary Fig. 10 | Mechanical self-healing of OHGel. a, Comparison of OHGels with different PE/GY ratio. PE10/GY3 is free-standing yet barely heals due to the limited chain motion; PE/GY7 heals rapidly, but at a cost of becoming non-free-standing; PE10/GY5 occupies both good mechanical integrity and rapid self-healing capability. **b,** Mechanical self-healing behavior of the PE10/GY5 OHGel characterized by uniaxial tensile test. After bisecting, rejoining, and healing at room temperature for 15 minutes, the OHGel (PE10/GY5) could restore 96.5% stretchability and 100% ultimate tensile strength.

Self-healing mechanism of OHGel: OHGel is formed by gelating P($SPMA_{0.5}$-r-$MMA_{0.5}$) in a water/glycerol binary solvent. The amphiphilic polyelectrolyte comprises hydrophilic segments (SPMA) that dissolve in the polar solvent, and hydrophobic segments (MMA) that associate with each other to

minimize their exposure to water. Such hydrophobic interaction gives rise to reversible physical crosslinks, with hydrophobes constantly associating and disengaging in a dynamic equilibrium. The electromechanical self-healing of OHGel takes two steps. First, the solvent in bisected OHGel samples join immediately upon physical contact and thus form a connected pathway for ion transportation. Secondly, the free hydrophobes on the cut surfaces can reassociate, with the help of polymer chain diffusion, to regain mechanical strength at the cut. The autonomous self-healing efficiency of OHGel is determined by the crosslinking kinetics. A short crosslink lifetime and a high disassociation/reassociation constant is considered favorable for fast self-healing.

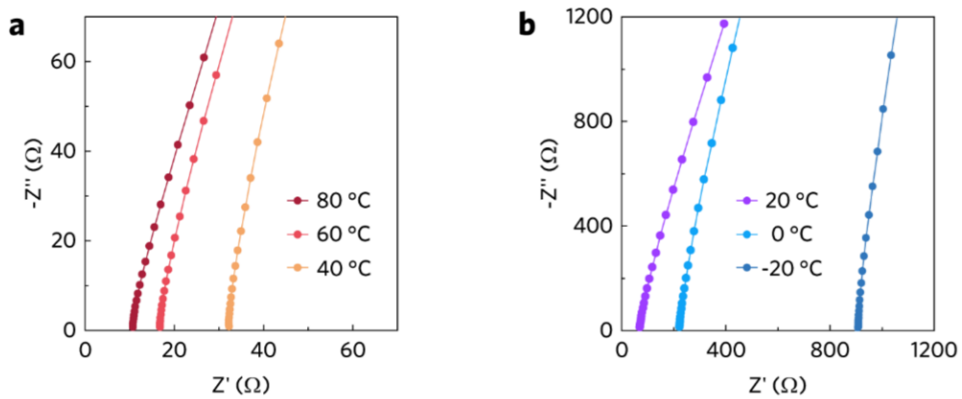

Supplementary Fig. 11 | Ionic conductivity of OHGel (PE10/GY5) at different temperature. Impedance spectra of OHGel are recorded at **(a)** 80 ℃, 60 ℃, 40 ℃ and **(b)** 20 ℃, 0 ℃, -20 ℃. Bulk resistance (R_i) of the OHGel sample can be estimated from the Nyquist plot by extrapolating its high frequency intercept on Z' axis. Ionic conductivity is calculated by $\sigma = d/(R_i \times A)$, where d and A are the thickness and the lateral area of the OHGel sample, respectively.

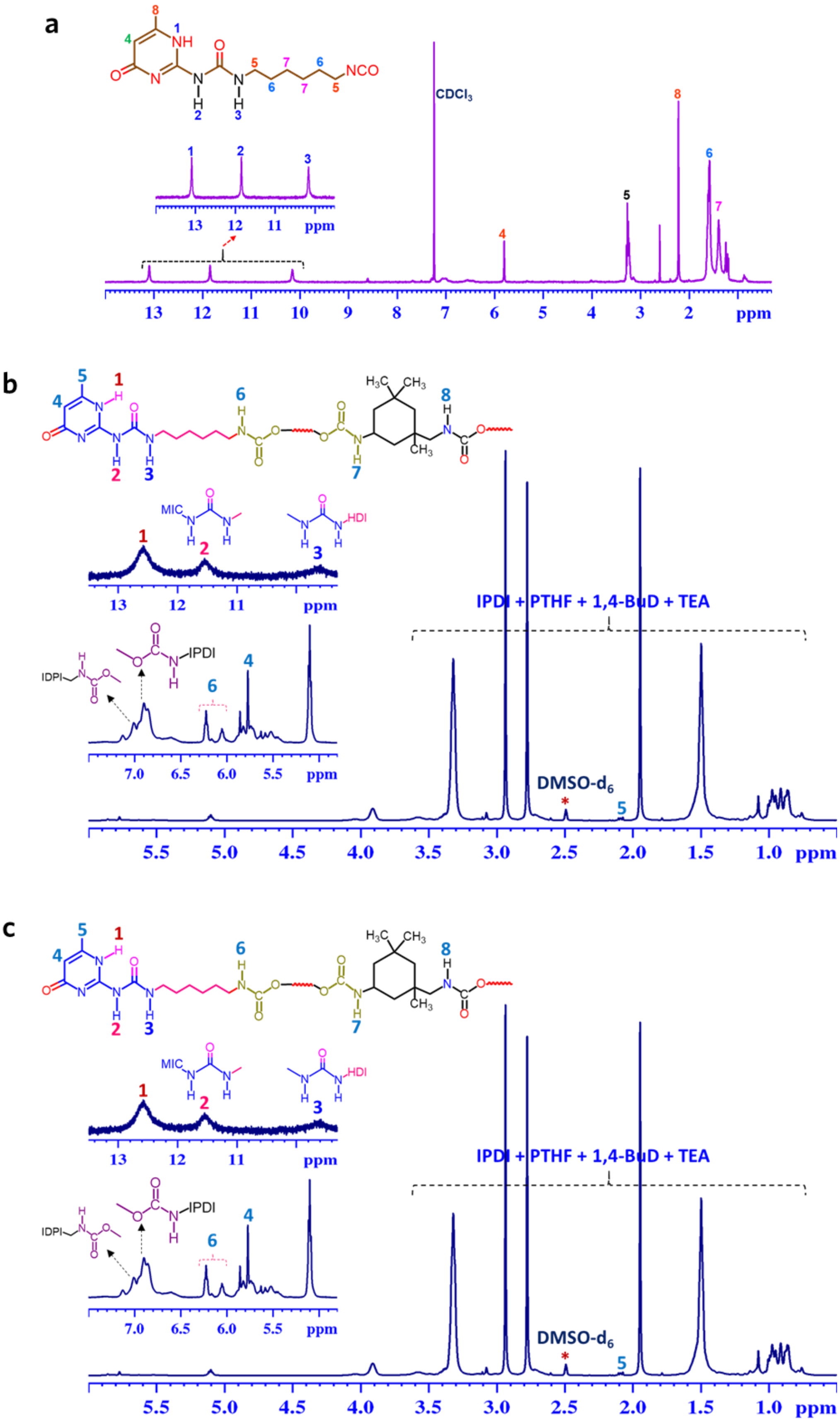
a
NH
NCO
CDCl$_3$
13 12 11 ppm
13 12 11 10 9 8 7 6 5 4 3 2 ppm
b
H_3C CH_3
CH_3
MIC
HDI
13 12 11 ppm
IPDI + PTHF + 1,4-BuD + TEA
IDPI
IPDI
7.0 6.5 6.0 5.5 ppm
DMSO-d$_6$
5.5 5.0 4.5 4.0 3.5 3.0 2.5 2.0 1.5 1.0 ppm
c
H_3C CH_3
CH_3
MIC
HDI
13 12 11 ppm
IPDI + PTHF + 1,4-BuD + TEA
IDPI
IPDI
7.0 6.5 6.0 5.5 ppm
DMSO-d$_6$
5.5 5.0 4.5 4.0 3.5 3.0 2.5 2.0 1.5 1.0 ppm

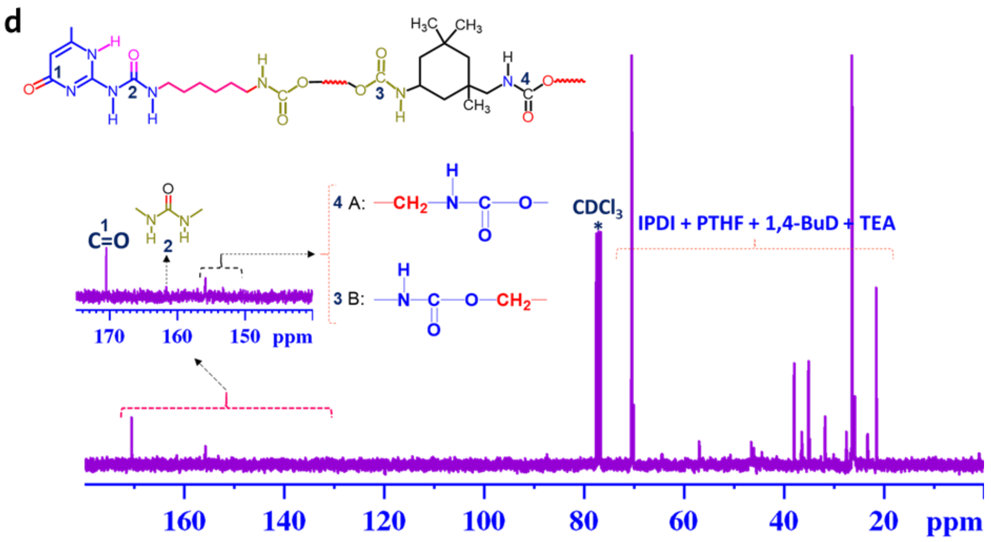

Supplementary Fig. 12 | NMR of SHPU and its precursors. a, ^{1}H-NMR of MIC. (400 MHz, CDCl$_3$, 25 °C, δ/ppm): 10.68 (s, 1H), 6.48 (s, 1H), 5.37 (s, 1H), 1.97 (s, 3H). **b,** ^{1}H-NMR of UPy-NCO. (400 MHz, CDCl$_3$, 25 °C, δ/ppm): 13.08 (s, 1H, CH$_3$-C-N$\underline{\text{H}}$), 11.83 (s, 1H, CH$_2$-NH-(C=O)-N$\underline{\text{H}}$), 10.15 (t, 1H, CH$_2$-N$\underline{\text{H}}$-(C=O)-NH), 5.79 (s, 1H, C$\underline{\text{H}}$=C-CH$_3$), 3.26 (m, 4H, NH-(C=O)-NH-C$\underline{\text{H}}_2$ + C$\underline{\text{H}}_2$-NCO), 2.22 (s, 3H, C$\underline{\text{H}}_3$), 1.59 (m, 4H, N-CH$_2$-C$\underline{\text{H}}_2$), 1.38 (m, 4H, CH$_2$-CH$_2$-C$\underline{\text{H}}_2$-C$\underline{\text{H}}_2$-CH$_2$-CH$_2$-NCO).

c, ^{1}H-NMR of SHPU. (400 MHz, DMSO-d$_6$, 25 °C, δ/ppm): 12.57 (m, 1H, CH$_3$-C-N$\underline{\text{H}}$), 11.52 (m, 1H, CH$_2$-NH-(C=O)-N$\underline{\text{H}}$), 9.62 (m, 1H, CH$_2$-N$\underline{\text{H}}$-(C=O)-NH), 7.32 (m, 2H, -N$\underline{\text{H}}$-(C=O)-O-CH$_2$-), 6.88 (m, 4H, -CH$_2$-N$\underline{\text{H}}$-(C=O)-O-), 6.21 (t, 3H, CH$_2$-N$\underline{\text{H}}$-(C=O)-NH-C-CH$_3$), 6.03 (m, 3H, CH$_2$-NH-(C=O)-N$\underline{\text{H}}$-C-CH$_3$), 5.75 (s, 2H, C$\underline{\text{H}}$=C-CH$_3$), 4.01 (-C-C$\underline{\text{H}}_2$-O-(C=O)-NH-), 3.22 (t, 3H, CH$_2$-C$\underline{\text{H}}_2$-O-(C=O)-NH-), 3.07 (-C$\underline{\text{H}}_2$ in IPDI), 2.33 (s, 3H, CH$_3$ in UPy), 2.93 (-C$\underline{\text{H}}_2$-CH$_2$-CH$_2$-CH$_2$- in PTMG, TEA), 1.95, 1.48 (-C$\underline{\text{H}}_2$- in IPDI; -CH$_2$-C$\underline{\text{H}}_2$-C$\underline{\text{H}}_2$-CH$_2$- in PTMG and 1,4-BuD), 1.09 (-CH$_2$- in IPDI), 1.01 (-CH$_2$ in IPDI; -CH$_3$ in IPDI), and 0.87 (-C(CH$_3$)$_2$ in IPDI). With the incorporation of UPy end groups, large down field chemical shifts are observed at δ = 12.57, 11.52, and 9.62 ppm as the evidence for UPy dimerization. The peaks at 7.32 and 6.88 ppm are assigned to urethane groups (-NH-(C=O)-O), indicating that IPDI has fully reacted with PTHF, 1,4-BuD, and TEA.

d, ^{13}C NMR of SHPU. (400 MHz, DMSO-d$_6$, 25 °C, δ/ppm): 169.80, 158.70, 155.18, 154.41, 68.83, 64.99, 63.14, 56.01, 56.16, 42.51, 38.31, 37.18, 33.74, 32.81, 31.44, 28.01, 27.22, 24.77, 22.17, 20.05, 18.45. The peaks appearing at 158.70 and 155.18 ppm are assigned to the secondary and primary urethane groups (-NH-(C=O)-O), respectively.

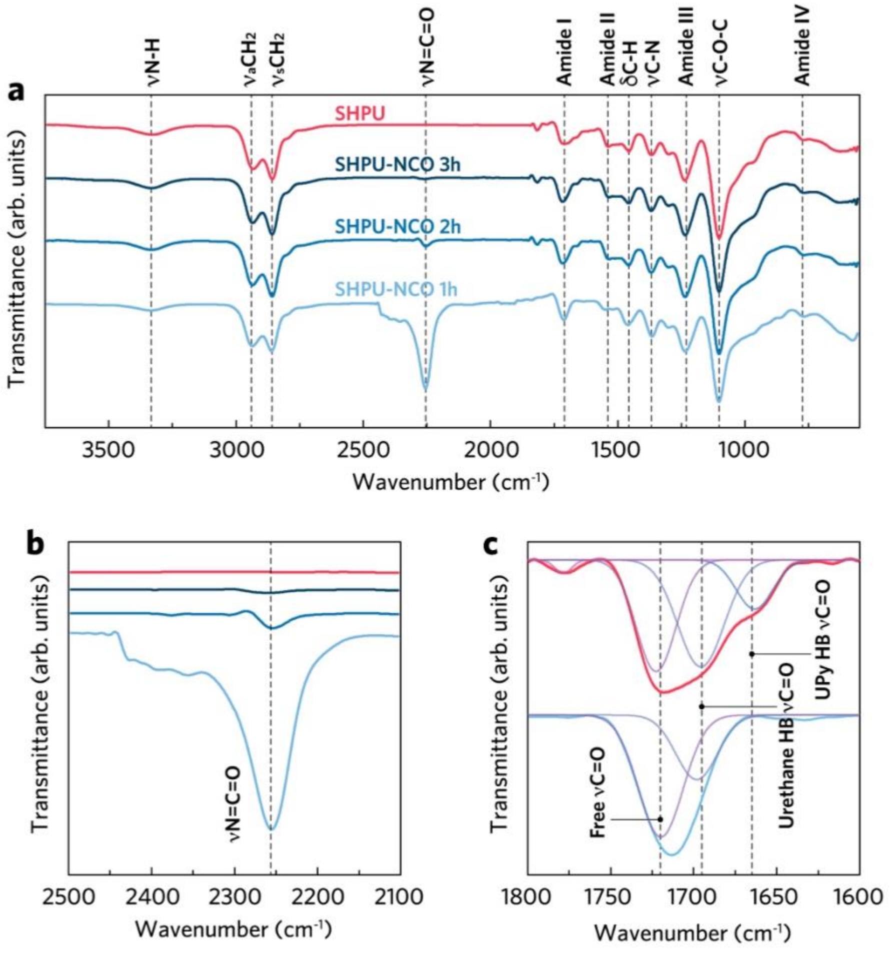

Supplementary Fig. 13 | FTIR characterization revealing the hierarchical H-bonding in SHPU. a, FTIR spectra of fully cured SHPU (red) and the SHPU prepolymer solution during synthesis (Step III, blue) of different reaction duration. **b,** Magnified spectra from 2500 cm^{-1} to 2100 cm^{-1} featuring the *v*(N=C=O) peak. The prominent peak of isocyanate group (-NCO) at 2256 cm^{-1} gradually disappeared as the reaction continued, and finally diminished in the spectrum of SHPU, suggesting a complete consumption of -NCO. **c,** Deconvoluted FTIR spectrum of SHPU in *v*C=O region unravelling various C=O stretching modes. HB = hydrogen bonded. The peak at 1715 cm^{-1} in the prepolymer spectrum represents a collection of free C=O stretching from urethane and UPy group; in SHPU spectrum, the 1720 cm^{-1} peak is assigned to a mixed contribution from free urethane C=O and free amide C=O in UPy, while H-bonded urethane C=O and H-bonded UPy C=O appear at 1695 cm^{-1} and 1665 cm^{-1}, respectively.

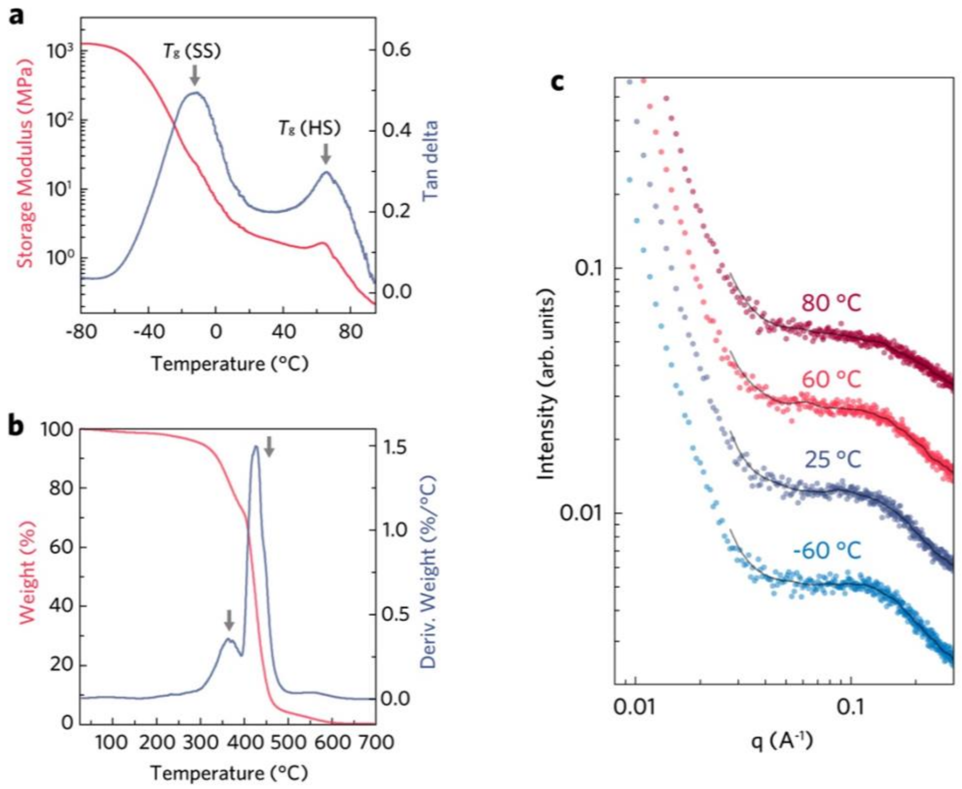

Supplementary Fig. 14 | Evidence of phase separation in SHPU. a, Storage modulus (*G*') and loss factor (Tan $\delta = G''/G'$) of SHPU recorded in a temperature range from -80 to 100 ℃. Two distinct glass transition temperatures are observed at -12 ℃ and 65 ℃, respectively. At room temperature, soft segments are above their T_g (-12 ℃) and confer high chain mobility for reversible H-bonding formation, whereas hard segments stay below their T_g (65 ℃) and thus reinforce the polymeric network. **b,** TGA trace and its first derivative of SHPU recorded in a temperature range from 25 to 700 ℃. Two decomposition temperatures observed at 365 ℃ and 426 ℃ also proves the biphasic composition of SHPU. **c,** SAXS results of SHPU under a wide range of temperatures. The intensive and broad scattering peaks detected under -20 ℃, 25 ℃, and 60 ℃ suggest the spherical geometry and uniform dispersion of hard domains (packed IPDI-urethane groups) in the soft matrix. The reduced peak intensity at 80 ℃ is attributed to the glass transition in hard domains.

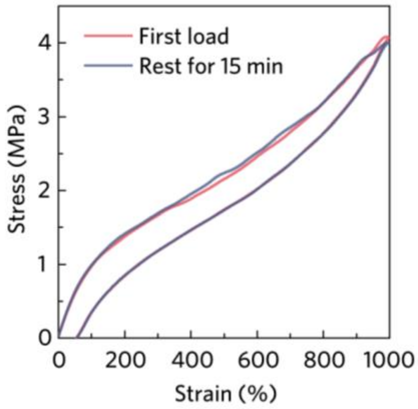

Supplementary Fig. 15 | Stress-strain loops of SHPU recorded through the first and the second loading cycle with 15 min rest in between. The almost identical loops suggest a complete recovery from cyclic fatigue.

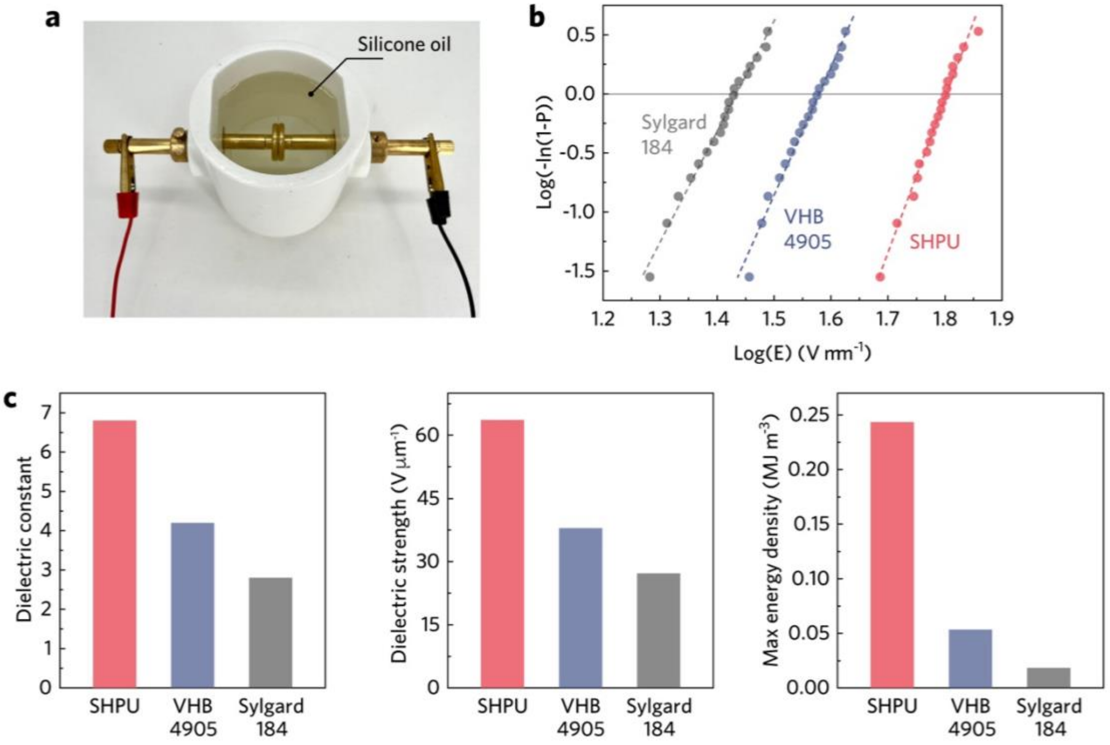

Supplementary Fig. 16 | Dielectric property of the elastomers. a, The setup for dielectric breakdown test. **b,** Weibull distribution of dielectric breakdown of SHPU, VHB 4905, and Sylgard 184. **c,** comparison of dielectric constant (100 Hz), dielectric strength, and maximum electrostatic energy density of SHPU, VHB 4905, and Sylgard 184.

Evaluation of dielectric strength and energy density:

Dielectric strength (breakdown field) of the elastomers was evaluated by following ASTM-D3755. As shown in Supplementary Fig. 16a, a specimen (thickness ≈ 200 μm) was mounted between two opposing brass probes (diameter = 25 mm) with the testing cup filled with silicone oil to prevent flashover and premature breakdown. A Hipot tester (Chroma 9056) was employed to supply DC potential at a ramping rate of 500 V s^{-1} with 0.1 mA cutoff current that defines a breakdown event. The breakdown voltage (U_B) and SHPU film thickness (d) were recorded to calculate breakdown field ($E_B = U_B/d$). The results were analyzed by fitting into the two parameter Weibull distribution function:

$$P(E,\beta) = 1 - e^{-(E/E_B)^{\beta}}$$

where P is the cumulative probability of dielectric breakdown occurring at electric field equal to or below E, β is the shape parameter (Weibull modulus) that describes the scattering of data, and E_B is the characteristic breakdown strength at a cumulative failure probability of 63.2%. To find E_B, the function was rearranged and plotted as log(-ln(1-P)) against E (Supplementary Fig. 16b). When P = 0, $E = E_B$. The characteristic dielectric strength of SHPU was determined to be 63.6 V μm^{-1}. This value is comparable with some commercial TPUs, such as Elastollan® 1185A10 (product of BASF, E_B = 88.6 V μm^{-1})[27]. Based on the same method, the dielectric strength of VHB 4905 and Sylgard 184 was measured to be 37.9 V μm^{-1} and 27.2 V μm^{-1} and, respectively. The Weibull modulus (β) of SHPU, VHB 4905, and Sylgard 184 is fitted to be 13.0, 10.9, and 9.4, respectively. The maximum electrostatic energy density for SHPU, VHB 4905, and Sylgard 184 is calculated to be 0.243, 0.053, and 0.018 MJ m^{-3}, respectively. SHPU exhibits 12 times higher energy density than Sylgard 184.

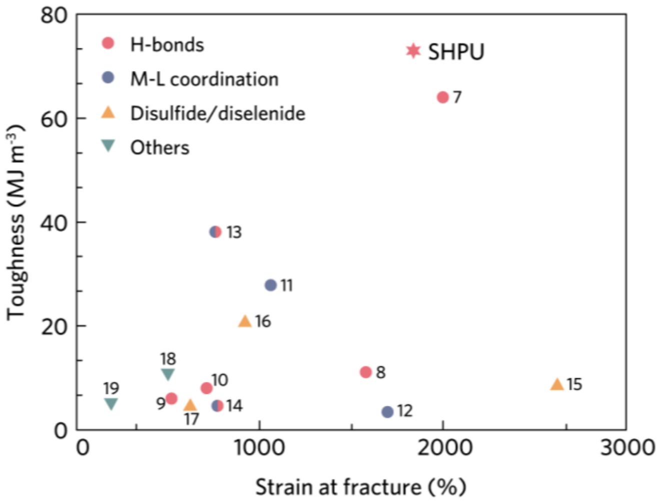

Supplementary Fig. 17 | Ashby plot comparing the toughness and fracture strain (after self-healing) of various room-temperature self-healable elastomers. Reference numbers are annotated near the data points.

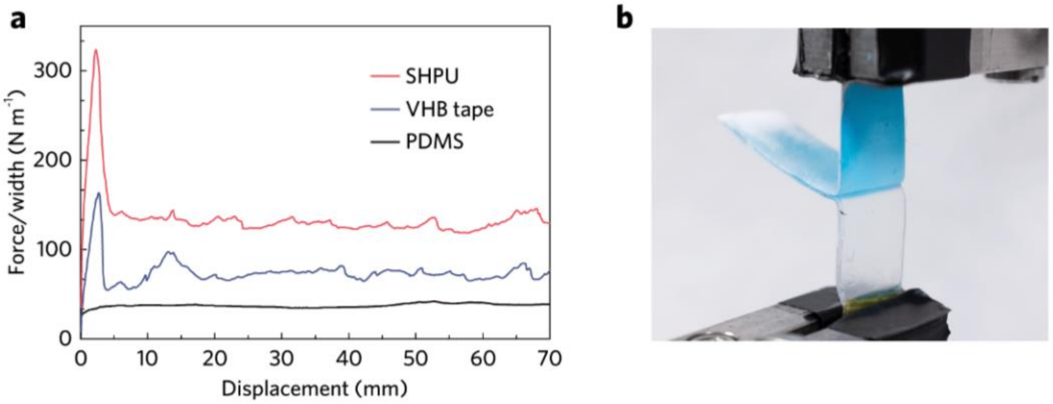

Supplementary Fig. 18 | T-peel tests following ASTM D1876. a, Recorded steady-state peeling force per unit width as a function of peeling distance for various OHGel-elastomer hybrids. **b,** Photograph showing the T-peel test condition.

In this testing setup, nylon membrane filter is utilized as the backing for OHGel out of two major reasons: (1) nylon membrane is mechanically stiff (Young's modulus > 1 GPa)[28] so that it can hold the integrity of OHGel layer during T-peel tests; (2) the hydrophilic and microporous nylon membrane allows OHGel to infiltrate so that it ensures a reliable bonding. Additionally, 3M Scotch tape serves as the backing for

the elastomers to prevent unwanted elongation in SHPU along the peeling direction. Interfacial toughness of the hybrids is derived from the as measured steady-state peeling force per unit width (Supplementary Fig. 18a) according to:

$$Interfacial\ toughness = \frac{2F_{\mathrm{p}}}{w}$$

where F_{p} is the averaged force at plateau and w is the width of the testing specimens. Compared with covalently bonded gel-elastomer systems, the substantial adhesion at OHGel-SHPU interface is autonomously established under ambient condition without requiring any chemical treatment, coupling agent or environmental stimulus.

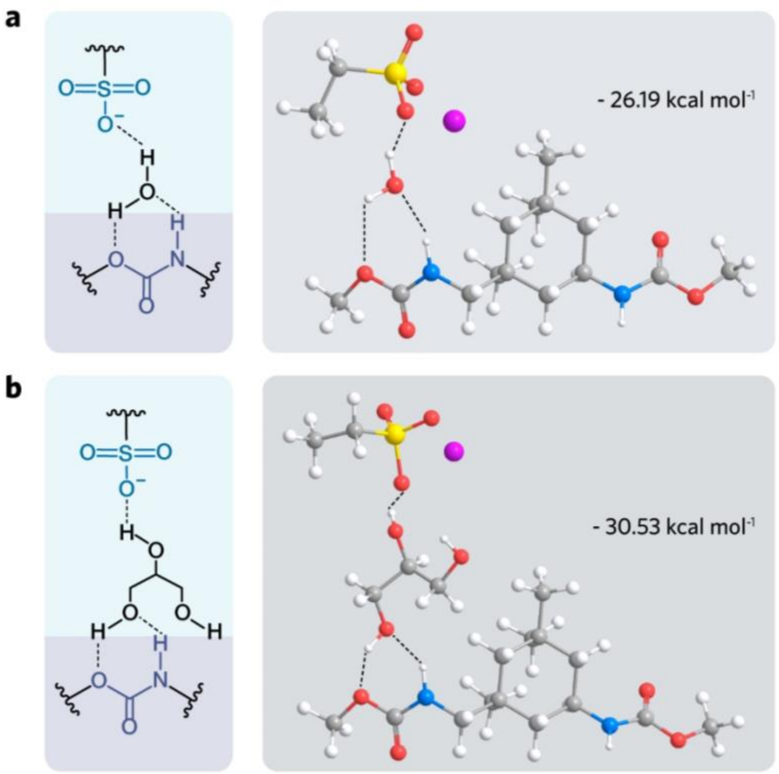

Supplementary Fig. 19 | DFT analysis of the OHGel-SHPU interfacial bonding. a, Water molecule-bridged ion-dipole interaction. **b,** Glycerol molecule-bridged ion-dipole interaction.

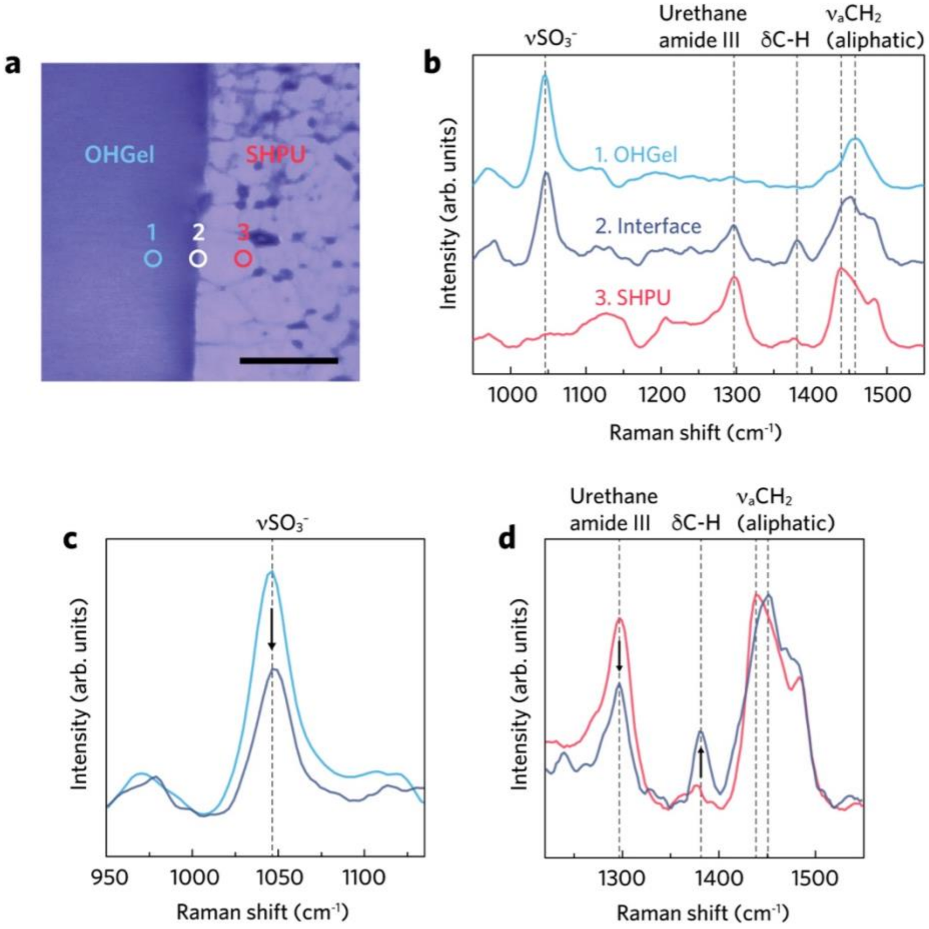

Supplementary Fig. 20 | Confocal Raman microscopy investigating OHGel-SHPU interfacial bonding. a, Optical microscopic image of the OHGel-SHPU hybrid indicating the locations where Raman spectra are collected. The 633 nm Raman laser in focus has a light spot diameter as small as 11 μm for localized Raman examination. Scale bar, 50 μm. **b,** Raman spectra collected on OHGel, SHPU, and at the OHGel-SHPU interface. **c,** Magnified spectral view focusing on the *v*SO^{3-} peaks. **d,** Magnified spectral view focusing on the characteristic peaks in urethane groups.

The Raman spectra were normalized to the bands around 1445 cm^{-1} as they are attributed to C-H stretching in aliphatic segments[29] which are not involved in the formation of interfacial H-bonding. As manifested by the band intensity changes at the interface compared with pure materials, noncovalent association modalities could be proposed between OHGel and SHPU. SO^{3-} vibration band[30] originated from the ionic sites in P($SPMA_{0.5}$-r-$MMA_{0.5}$) appears at 1046 cm^{-1} in both OHGel and the interface spectrum. The lower band intensity at interface (Supplementary Fig. 20c) suggests a dynamic equilibrium

in SO^{3-} between the dissociation from hydration in bulk OHGel and the reassociation with proton donors from the opposing SHPU surface. Comparing SHPU and interface spectra, the intensity increase of C-H bending (associated with urethane -N)[31] at 1376 cm^{-1} is detected, whereas the band of urethane amide III[32,33] at 1300 cm^{-1} decreases (Supplementary Fig. 20d). The drastic peak ratio variations are due to the conformational changes in urethane groups upon contact with OHGel.

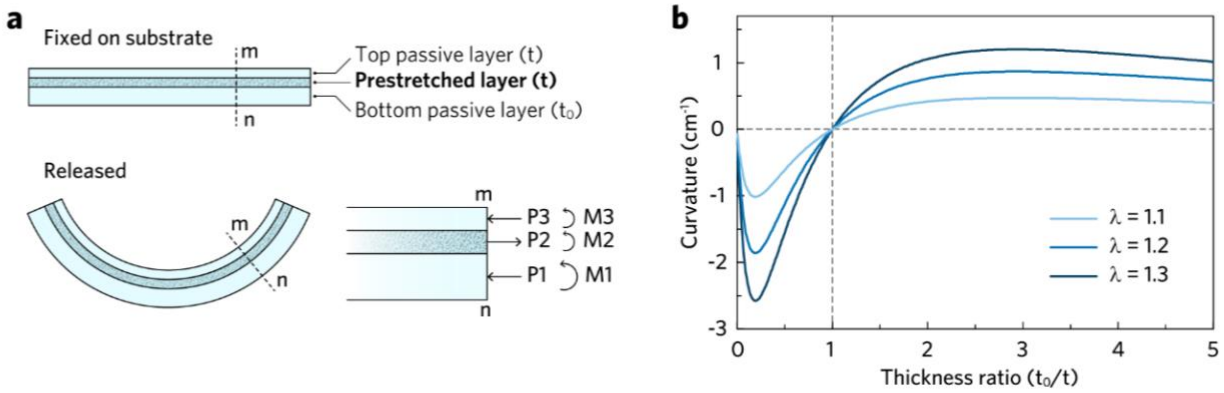

Supplementary Fig. 21 | Timoshenko analysis predicting the bending curvature of the unimorph DEA at rest. a, Schematic illustrations of the SHPU trilayer model with internal forces and bending moments being annotated. **b,** Predicted curvature as a function of pre-stretch (λ) and thickness ratio (t/t_0).

The unimorph DEA in our gripper adopts a dielectric elastomer minimum energy structure (DEMES) to introduce a curvature at rest. Specifically, elastic energy stored in a prestretched SHPU thin film will partially transfer to the bonded passive layers as bending energy upon release, and the minimization of total free energy give rise to a controllable out-of-plane structure. The bending curvature is determined by the mechanical properties of SHPU, the geometrical parameters such as the thickness difference between top and bottom passive layers, and the degree of prestretch ($\lambda = 1 + \varepsilon$). DEMES deformation induced by prestretch can be predicted using Timoshenko analysis,[34] which was established a century ago and has been widely employed to describe the actuating behavior of bilayer actuators based on multifarious mechanisms.[35-37] To simplify the derivation process, we omit the influence of OHGel

electrodes in the system considering their compliance and low thickness. It is considered that such simplification leads to acceptable deviation from real conditions.

The SHPU trilayer illustrated in Supplementary Fig. 21a consists of a prestretched middle layer (thickness t), a passive top layer (thickness t), and a passive bottom layer (thickness t_0), which are tightly bonded without interfacial sliding. The width of the beam is normalized to unity. While internal stress builds up in the middle layer due to prestretch, the constrains from the passive layers restrict its lateral retraction, and the difference in top and bottom layer thickness ($t_0 > t$) directs the beam to bend concave upward. The resultant axial forces (P_1, P_2, P_3) and bending moments (M_1, M_2, M_3) are annotated in Supplementary Fig. 21a (bottom right).

We examine the left-hand side of the cross section ***mn***, which should remain planar and normal to the longitude axis constantly according to the theorem of pure bending.[38] As the beam perceives no external force or moment, the net force and net moment at ***mn*** should be zero. Summing axial forces and adding up bending moments about the cut, we get

$$\sum P = 0 \qquad P_1 + P_3 - P_2 = 0 \tag{1}$$

$$\sum M = 0 \qquad P_1 \frac{t_0 + t}{2} - P_3 t = M_1 + M_2 + M_3 \tag{2}$$

From moment-curvature relationship we have

$$M_1 = EI_0\kappa \qquad M_2 = M_3 = EI\kappa$$

where E is the Young's modulus of SHPU; κ is beam curvature; I and I_0 are the area moment of inertia of each layer's cross-section. Substitute the above equations into (2), we obtain

$$P_1 \frac{t_0 + t}{2} - P_3 t = E\kappa(I_0 + 2I) \tag{3}$$

Besides, geometrical constraint requires that the strain levels in the prestretched layer and each passive layer should be identical at their bearing interfaces:

$$\varepsilon' + \frac{P_2}{Et} + \frac{t\kappa}{2} = -\frac{P_1}{Et_0} - \frac{t_0\kappa}{2} \tag{4}$$

$$\varepsilon' + \frac{P_2}{Et} - \frac{t\kappa}{2} = -\frac{P_3}{Et} - \frac{t\kappa}{2} \tag{5}$$

The equivalent strain ε', defined as $\varepsilon' = -\varepsilon/(1+\varepsilon)$, describes the relative change in length when the prestretched layer is released back to the stress-free status. By replacing

$$n = \frac{t_0}{t} \qquad I_0 = \frac{{t_0}^3}{12} \qquad I = \frac{t^3}{12}$$

and solving the matrix (1) (3) (4) (5), we get the curvature-thickness relationship of our SHPU trilayer as

$$\kappa = \frac{6\varepsilon'}{t}\frac{1-n}{(n+2)(n^2+12n+12+2/n\,)} \tag{6}$$

The above equation clearly reveals that the bending of SHPU trilayer at minimal free energy is predominantly determined by prestretch and the thickness ratio between t_0 and t (Supplementary Fig. 21b). The parameters utilized for the fabrication of our unimorph DEA is as follow: n = t_0/t = 80/60 ≈ 1.33; λ = 1.2 (ε = 0.2).

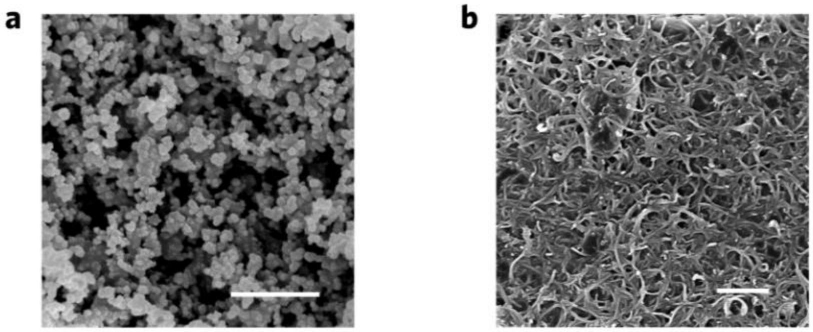

Supplementary Fig. 22 | a, SEM image of the silica nanoparticle assembly spray coated on a SHPU substrate. Scale bar, 500 nm. **b,** SEM image of the nanoporous carbon composite comprising CNT and graphite as conductive fillers, and SEBS as elastomeric binder. Scale bar, 1 μm.

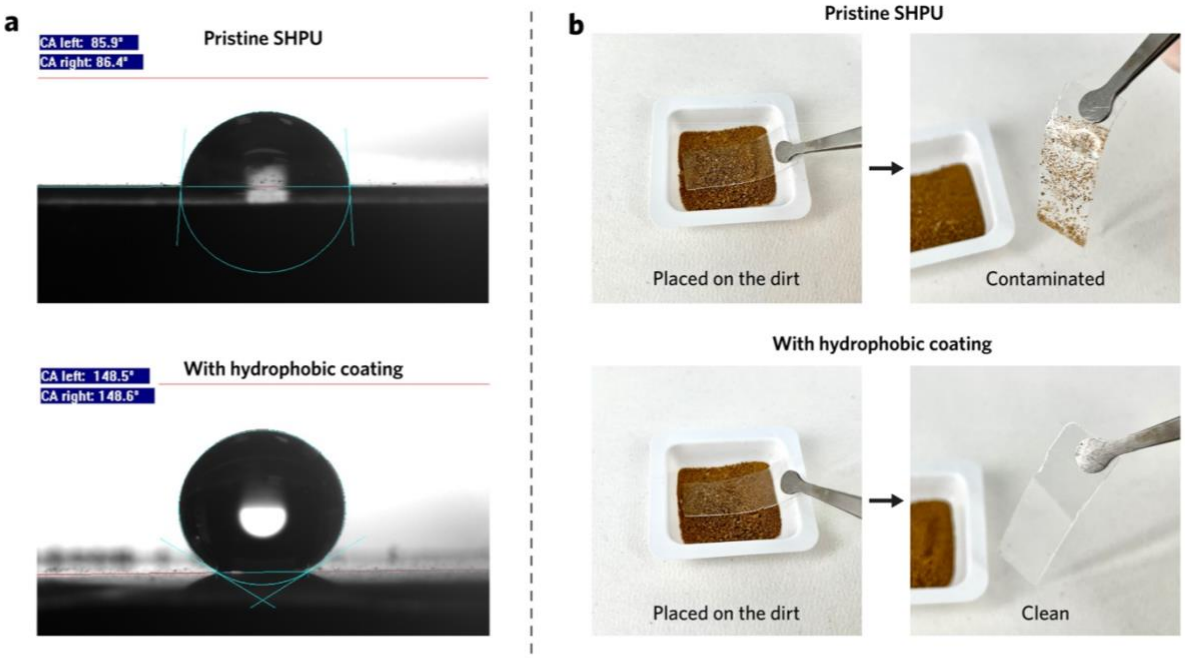

Supplementary Fig. 23 | Self-cleaning capability of the SHPU contact layer with hydrophobic coating. a, Water contact angle on pristine SHPU and SHPU coated with silica nanoparticles. **b,** SHPU membranes before and after contacting the dust. The pristine SHPU collected a lot of dust due to its inherent tackiness, whereas the one with hydrophobic coating maintained clean.

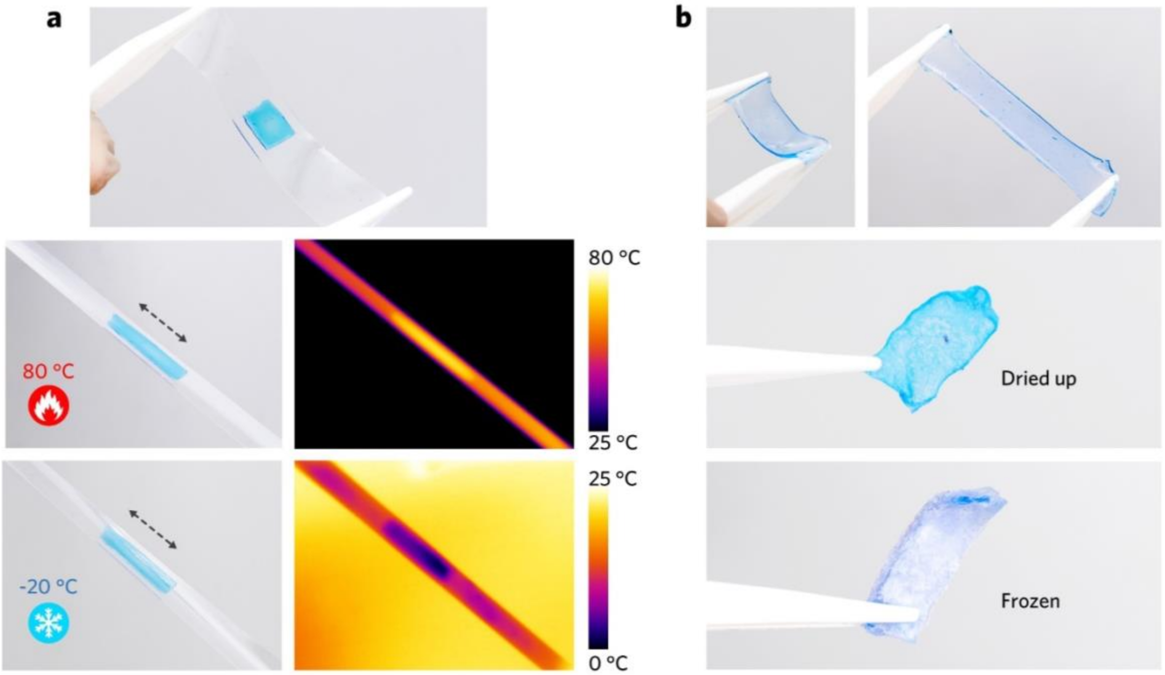

Supplementary Fig. 24 | Photographs contrasting the elastic stability between OHGel-SHPU composite and polyacrylamide (PAAm) hydrogel under extreme temperatures. a, The OHGel-SHPU composite maintained elastic after being stored in an 80 ℃ oven or a -20 ℃ fridge for 24 h. **b,** The PAAm hydrogel dried up at 80 ℃ and frozen into an ice-like solid at -20 ℃.

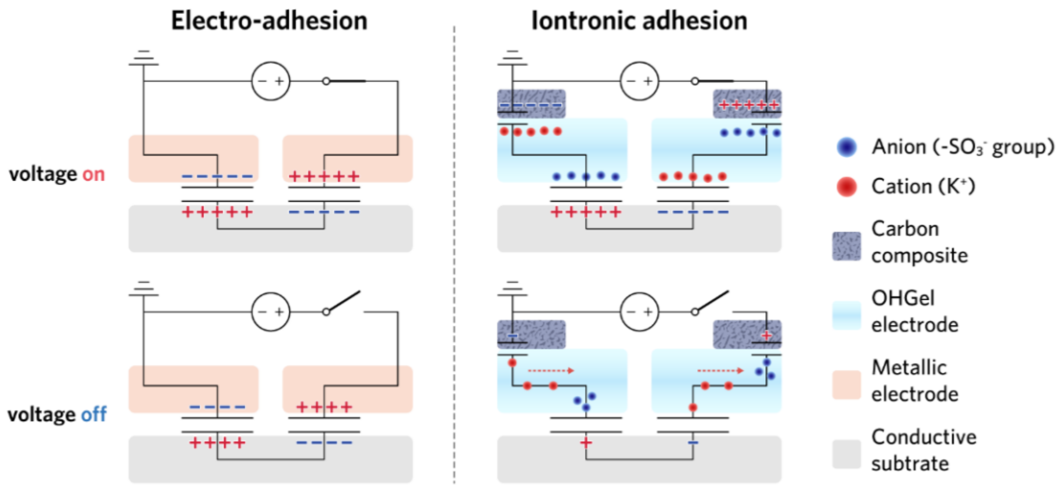

Supplementary Fig. 25 | Schematic illustrations revealing the discrepant adhering and detaching mechanisms between electroadhesion and iontronic adhesion. The dielectric layers that encapsulate the electrodes and insulate them from the conductive substrates are not illustrated here for visual simplification.

In an electroadhesion patch, turning off the voltage supply does not lead to a prompt release as the net charges coupled across the patch-substrate interface will persist due to the slow self-discharging of dielectric capacitors (Supplementary Fig. 25, left). As for iontronic adhesion, the voltage drop across EDL capacitor is well below 1 V when 1 kV is applied to the entire iontronic circuit. When the small voltage drop across EDL is removed, charges coupled at EDL can relax transiently through a mixed self-discharging mechanism of leakage current, faradaic charge transfer, and charge redistribution.[39] Net ionic charges released from EDL would subsequently diffuse and neutralize the counterions in the polyelectrolyte matrix through an entropy-driven process, and thus reduce the electrostatic strength between OHGel electrodes and the opposing metallic surface (Supplementary Fig. 25, right).

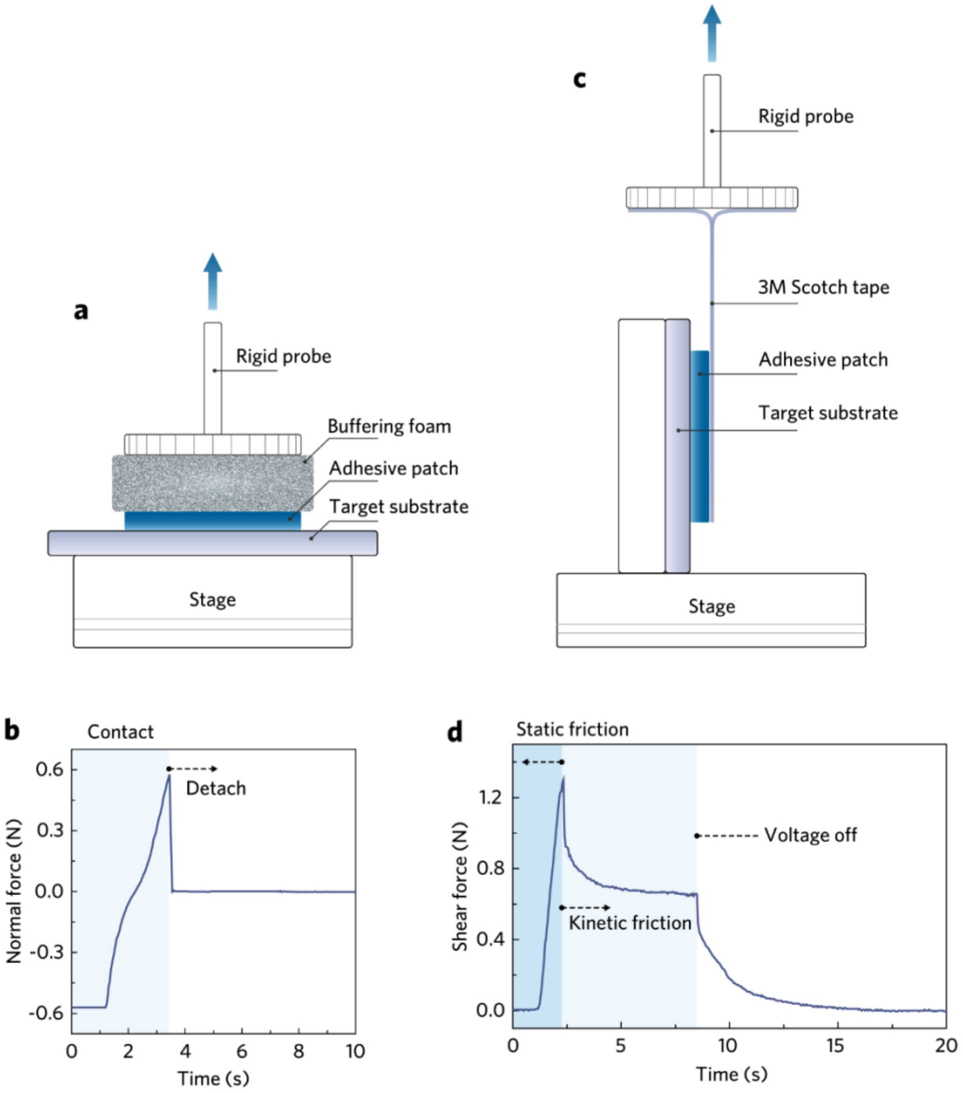

Supplementary Fig. 26 | Quantification of iontronic adhesion force. a, Customized testing setup for the measurement of normal adhesion force. **b,** Normal force recorded during a representative normal adhesion test on aluminum under 1 kV. The peak force recorded upon detachment is regarded as the measured normal force. **c,** Customized testing setup for the measurement of shear adhesion force. **d,** Shear force recorded during a representative shear adhesion test on aluminum under 1 kV. The maximum static frictional force is regarded as the measured shear adhesion force.

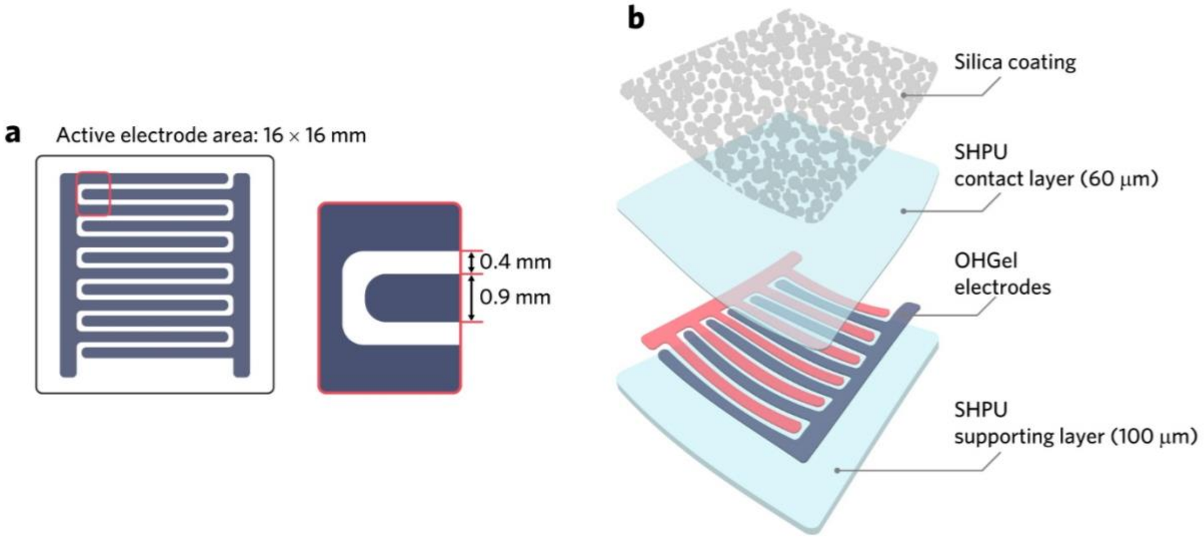

Supplementary Fig. 27 | Design of the OHGel-electroded iontronic-adhesive patch. a, Geometry of the OHGel electrodes in the iontronic-adhesive patch. The active electrode area is 16 × 16 mm. The width and pitch of the interdigitated electrodes are 0.9 mm and 0.4 mm, respectively. **b,** Exploded view of the layered structure of the iontronic-adhesive patch.

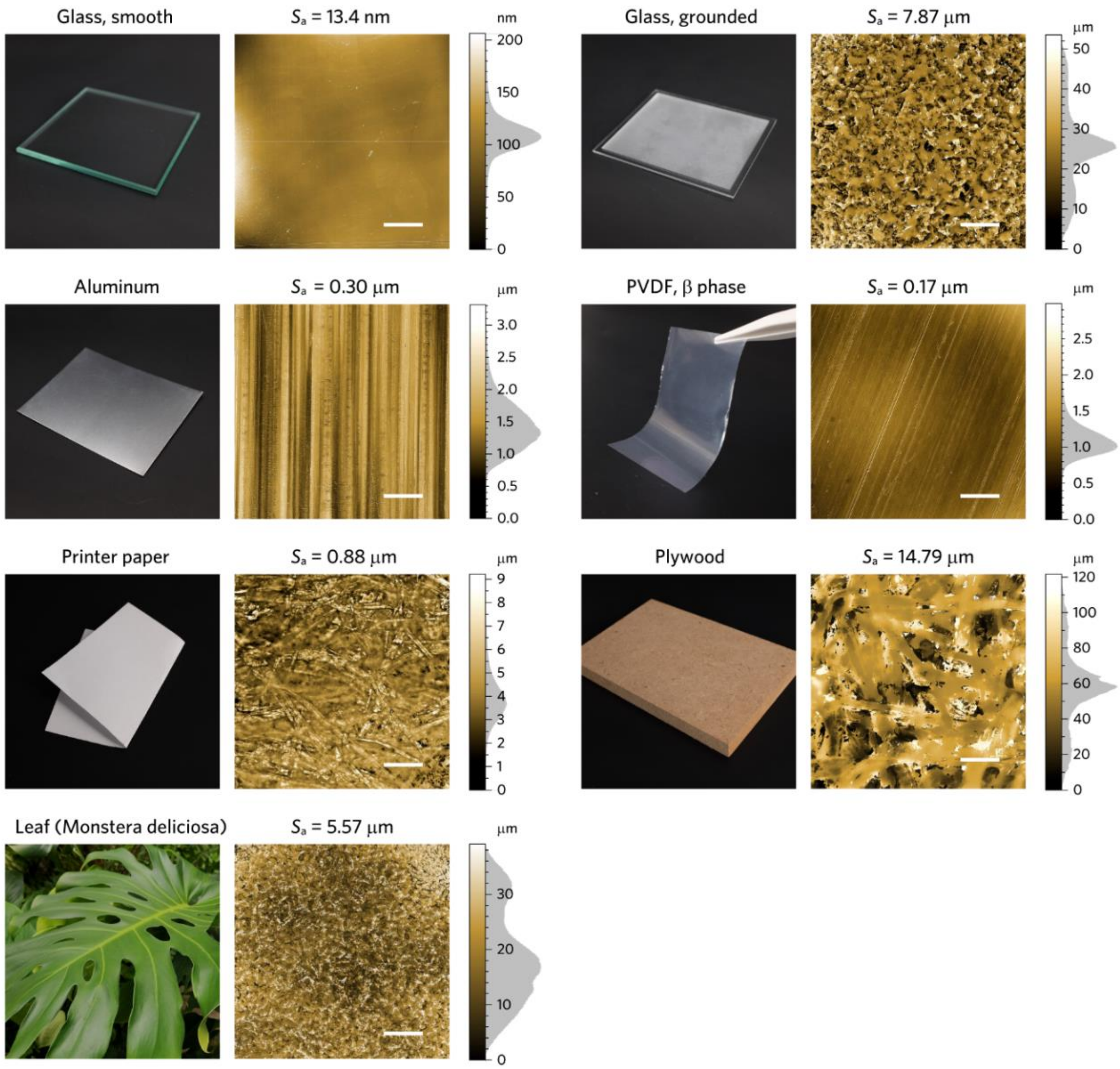

Supplementary Fig. 28 | Photograph, surface texture, and S_a of all the substrates employed in the characterization of iontronic adhesion force. Scale bars, 100 μm.

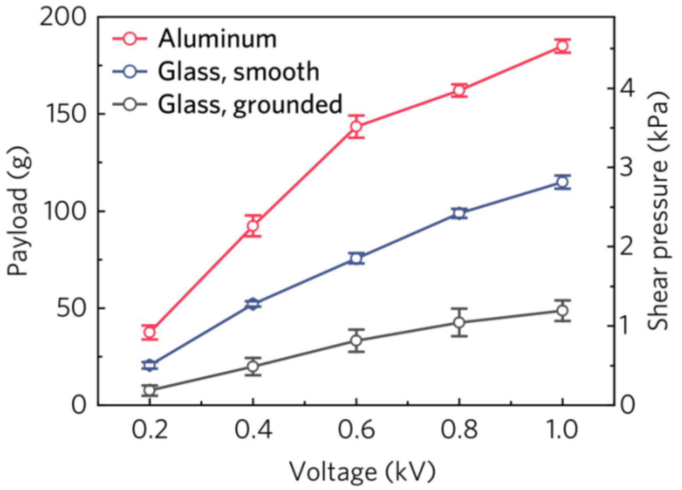

Supplementary Fig. 29 | Maximum payload (calculated) and shear pressure (measured) of the 4-fingered iontronic-adhesive soft gripper on different materials under a series of voltage input.

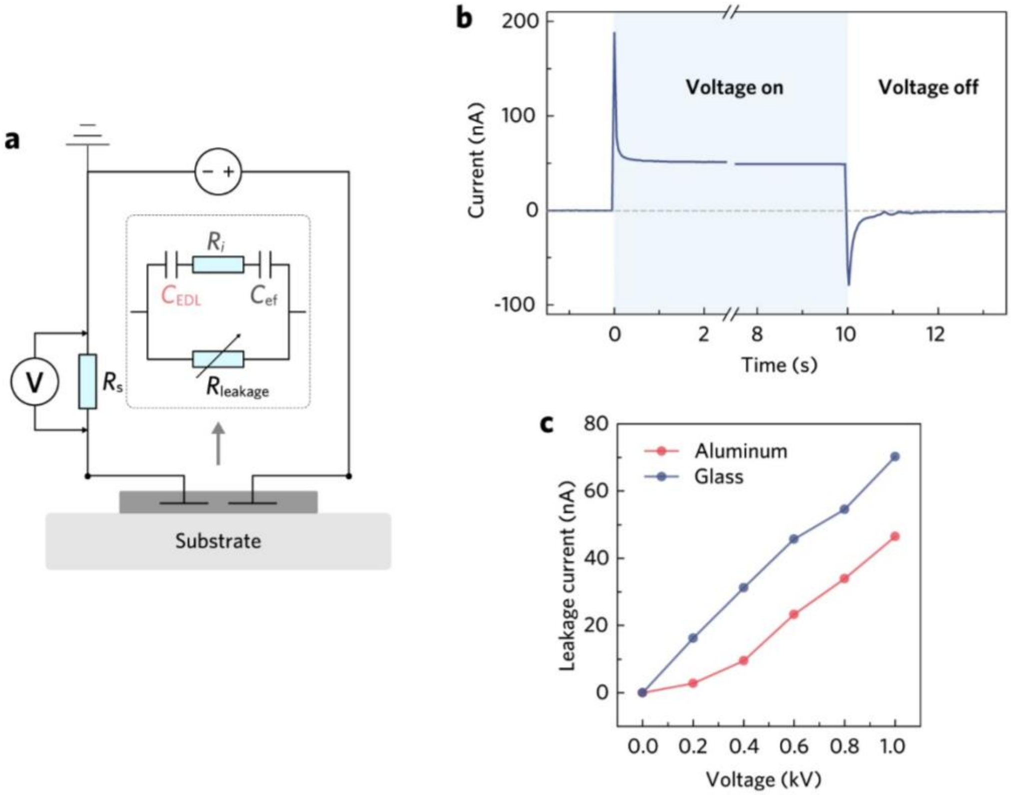

Supplementary Fig. 30 | Characterization of leakage current and power consumption. a, Testing setup that measures the charging, discharging, and leakage current of the iontronic-adhesive patches. **b,** Current recorded in the charging, steady, and discharging phases on aluminum at 1 kV. The small leakage current (~ 50 nA) suggests a low power consumption (~ 50 μW) of the patch. **c,** Leakage current of the patch on aluminum and glass (smooth) under different voltage bias.

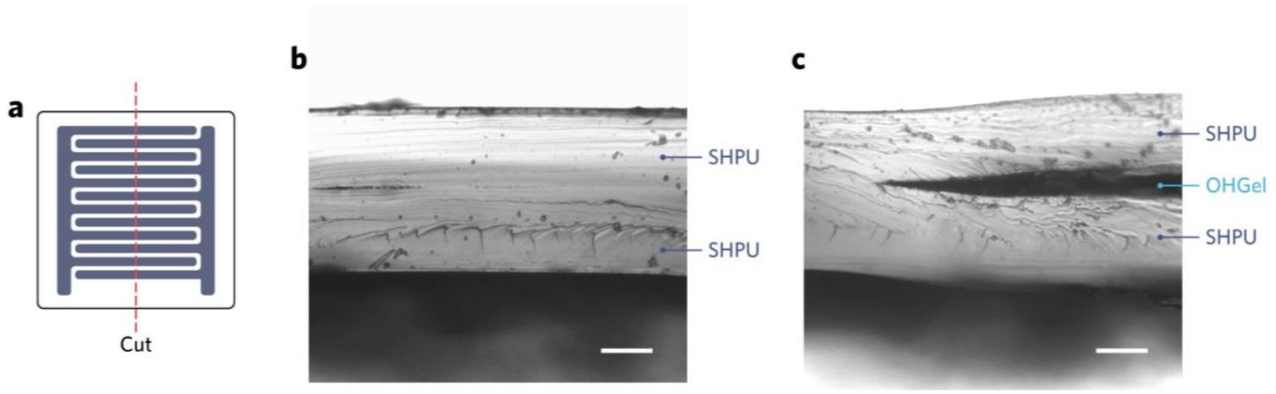

Supplementary Fig. 31 | Characterization of the cut surface morphology at the multilayered regions of an iontronic-adhesive patch. a, Schematic illustration showing the location of cut damage. **b,** Optical microscopic image revealing a SHPU-SHPU cross section. The interface between SHPU contact layer (top, 60 µm thick) and supporting layer (bottom, 100 µm thick) is barely visible, suggesting SHPU itself can form highly cohesive bonding between different layers. **c,** Optical microscopic image revealing a SHPU-OHGel-SHPU cross section. The cut surfaces of all three layers are left clean without plastic deformation.

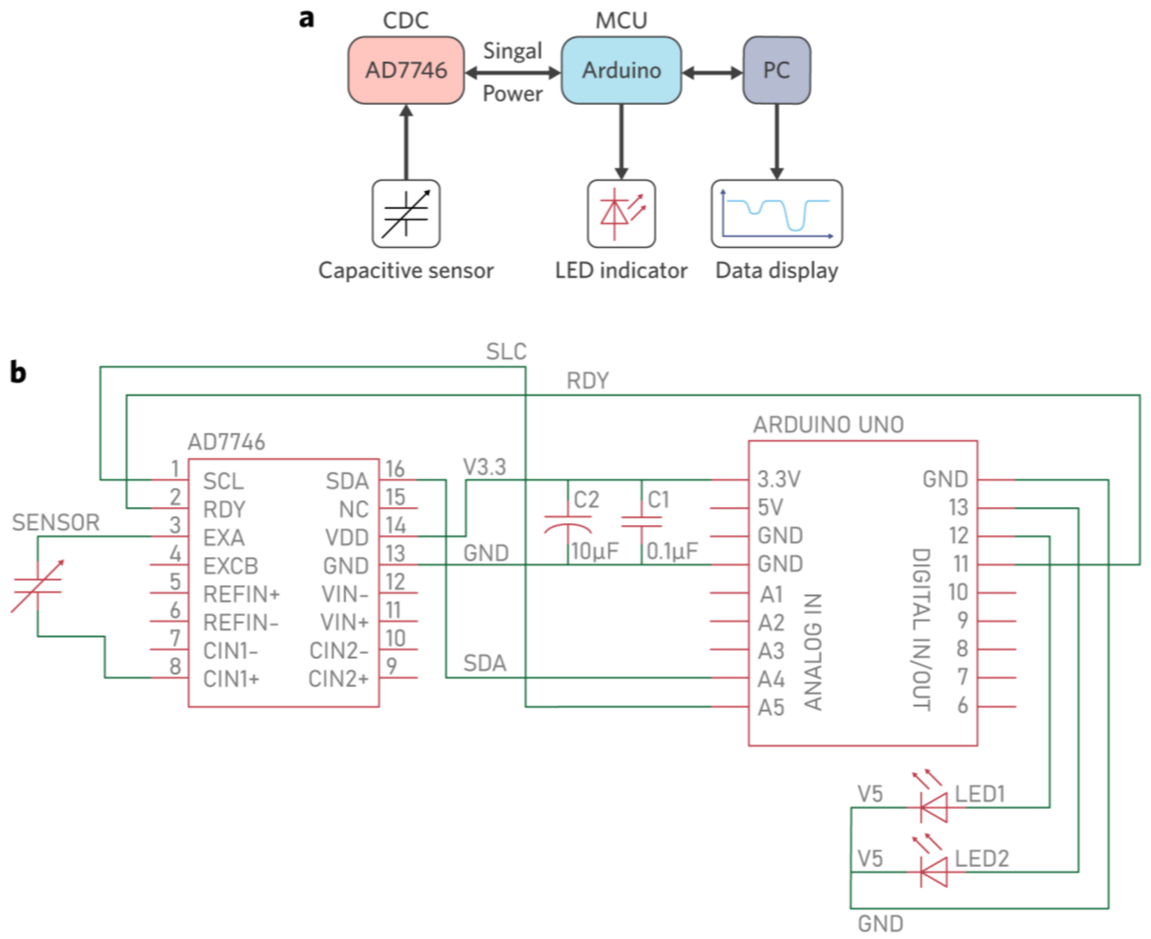

Supplementary Fig. 32 | Readout circuitry for exceptive sensing. a, Block diagram representing the communication between the components in the circuitry. **b,** Circuitry diagram detailing the wiring between the capacitive sensor (end effector in the iontronic gripper), the CDC module (AD7746), the microcontroller (Arduino), and the LEDs.

Supplementary Video Legends

Supplementary Video 1 | OHGel-SHPU bilayer under cyclic tensile test.

Supplementary Video 2 | Metallic cube handling and weightlifting of the iontronic gripper.

Supplementary Video 3 | Heat/freeze tolerance of the OHGel-SHPU material system.

Supplementary Video 4 | Iontronic gripper picking up an icing/scorching metallic cube.

Supplementary Video 5 | Iontronic gripper gently handling delicate objects.

Supplementary Video 6 | Iontronic gripper picking up flat/tiny objects.

Supplementary Video 7 | Two-fingered iontronic gripper driven by < 400 V voltage input.

Supplementary Video 8 | Exteroceptive sensing and real-time display of the iontronic gripper.

References

1. Lee, J. et al. Water-processable, stretchable, self-healable, thermally stable, and transparent ionic conductors for actuators and sensors. *Adv. Mater.* **32,** 1906679 (2020).

2. Folmer, B. J. B., Sijbesma, R. P., Versteegen, R. M., van der Rijt, J. A. J. & Meijer, E. W. Supramolecular polymer materials: Chain extension of telechelic polymers using a reactive hydrogen-bonding synthon. *Adv. Mater.* **12,** 874-878 (2000).

3. Perdew, J. P., Burke, K. & Ernzerhof, M. Generalized gradient approximation made simple. *Phys. Rev. Lett.* **77,** 3865 (1996).

4. Delley, B. An all-electron numerical method for solving the local density functional for polyatomic molecules. *The Journal of Chemical Physics* **92,** 508-517 (1990).

5. Grimme, S. Semiempirical gga-type density functional constructed with a long-range dispersion correction. *J. Comput. Chem.* **27,** 1787-1799 (2006).

6. Mishra, A. K., Chattopadhyay, D. K., Sreedhar, B. & Raju, K. V. S. N. Ft-ir and xps studies of polyurethane-urea-imide coatings. *Prog. Org. Coat.* **55,** 231-243 (2006).

7. Houston, K. R., Jackson, A.-M. S., Yost, R. W., Carman, H. S. & Sheares Ashby, V. Supramolecular engineering polyesters: Endgroup functionalization of glycol modified pet with ureidopyrimidinone. *Polymer Chemistry* **7,** 6744-6751 (2016).

8. Wang, D. et al. Transparent, mechanically strong, extremely tough, self-recoverable, healable supramolecular elastomers facilely fabricated via dynamic hard domains design for multifunctional applications. *Adv. Funct. Mater.* **30,** 1907109 (2019).

9. Kang, J. et al. Tough and water-insensitive self-healing elastomer for robust electronic skin. *Adv. Mater.* **30,** 1706846 (2018).

10. Hentschel, J., Kushner, A. M., Ziller, J. & Guan, Z. Self-healing supramolecular block copolymers. *Angew. Chem.* **124,** 10713-10717 (2012).

11. Chen, Y., Kushner, A. M., Williams, G. A. & Guan, Z. Multiphase design of autonomic self-healing thermoplastic elastomers. *Nat. Chem.* **4,** 467-472 (2012).

12. Lai, J. C. et al. Thermodynamically stable whilst kinetically labile coordination bonds lead to strong and tough self-healing polymers. *Nat. Commun.* **10,** 1164 (2019).

13. Li, C. H. et al. A highly stretchable autonomous self-healing elastomer. *Nat. Chem.* **8,** 618-624 (2016).

14. Zhang, L. et al. A highly efficient self-healing elastomer with unprecedented mechanical properties. *Adv. Mater.* **31,** e1901402 (2019).

15. Zhang, Q. et al. An elastic autonomous self-healing capacitive sensor based on a dynamic dual crosslinked chemical system. *Adv. Mater.***,** e1801435 (2018).

16. Rekondo, A. et al. Catalyst-free room-temperature self-healing elastomers based on aromatic disulfide metathesis. *Mater. Horiz.* **1,** 237-240 (2014).

17. Kim, S. M. et al. Superior toughness and fast self-healing at room temperature engineered by transparent elastomers. *Adv. Mater.* **30,** 1705145 (2018).

18. An, X. et al. Aromatic diselenide crosslinkers to enhance the reprocessability and self-healing of polyurethane thermosets. *Polymer Chemistry* **8,** 3641-3646 (2017).

19. Susa, A., Bose, R. K., Grande, A. M., van der Zwaag, S. & Garcia, S. J. Effect of the dianhydride/branched diamine ratio on the architecture and room temperature healing behavior of polyetherimides. *ACS Appl. Mater. Interfaces* **8,** 34068-34079 (2016).

20. Li, J., Ejima, H. & Yoshie, N. Seawater-assisted self-healing of catechol polymers via hydrogen bonding and coordination interactions. *ACS Appl. Mater. Interfaces* **8,** 19047-19053 (2016).

21. Shintake, J., Rosset, S., Schubert, B., Floreano, D. & Shea, H. Versatile soft grippers with intrinsic electroadhesion based on multifunctional polymer actuators. *Adv. Mater.* **28,** 231-238 (2016).

22. Cacucciolo, V., Shintake, J. & Shea, H. Delicate yet strong: Characterizing the electro-adhesion lifting force with a soft gripper. *2019 2nd IEEE International Conference on Soft Robotics (RoboSoft)*, 108-113 (IEEE, 2019).

23. Hwang, G., Park, J., Cortes, D. S. D., Hyeon, K. & Kyung, K.-U. Electroadhesion-based high-payload soft gripper with mechanically strengthened structure. *IEEE Transactions on Industrial Electronics* **69,** 642-651 (2021).

24. Xu, D. et al. Inkjet printing of polymer solutions and the role of chain entanglement. *J. Mater. Chem.* **17,** 4902 (2007).

25. Derby, B. Inkjet printing of functional and structural materials: Fluid property requirements, feature stability, and resolution. *Annu. Rev. Mater. Res.* **40,** 395-414 (2010).

26. Tekin, E., Smith, P. J. & Schubert, U. S. Inkjet printing as a deposition and patterning tool for polymers and inorganic particles. *Soft Matter* **4,** 703-713 (2008).

27. Yin, G. et al. Dielectric elastomer generator with improved energy density and conversion efficiency based on polyurethane composites. *ACS Appl. Mater. Interfaces* **9,** 5237-5243 (2017).

28. Inoue, A., Yuk, H., Lu, B. & Zhao, X. Strong adhesion of wet conducting polymers on diverse substrates. *Sci. Adv.* **6,** eaay5394 (2020).

29. Yang, Y. & Urban, M. W. Self-healing of glucose-modified polyurethane networks facilitated by damage-induced primary amines. *Polymer Chemistry* **8,** 303-309 (2017).

30. Edwards, H. G. M., Brown, D. R., Dale, J. A. & Plant, S. Raman spectroscopy of sulfonated polystyrene resins. *Vib. Spectrosc* **24,** 213-224 (2000).

31. Yang, Y. & Urban, M. W. Self-repairable polyurethane networks by atmospheric carbon dioxide and water. *Angew. Chem. Int. Ed. Engl.* **53,** 12142-12147 (2014).

32. Phadke, A. et al. Rapid self-healing hydrogels. *Proc. Natl. Acad. Sci. U. S. A.* **109,** 4383-4388 (2012).

33. Parnell, S., Min, K. & Cakmak, M. Kinetic studies of polyurethane polymerization with raman spectroscopy. *Polymer* **44,** 5137-5144 (2003).

34. Timoshenko, S. Analysis of bi-metal thermostats. *J. Opt. Soc. Am. Rev. Sci. Instrum.* **11,** 233-255 (1925).

35. Shian, S., Bertoldi, K. & Clarke, D. R. Dielectric elastomer based "grippers" for soft robotics. *Adv. Mater.* **27,** 6814-6819 (2015).

36. Gao, D. et al. Photothermal actuated origamis based on graphene oxide-cellulose programmable bilayers. *Nanoscale Horiz* **5,** 730-738 (2020).

37. Hu, Y. et al. A graphene-based bimorph structure for design of high performance photoactuators. *Adv. Mater.* **27,** 7867-7873 (2015).

38. Gere, J. M. & Goodno, B. J. *Mechanics of materials* (Brooks Cole, 2001)

39. Lewandowski, A., Jakobczyk, P., Galinski, M. & Biegun, M. Self-discharge of electrochemical double layer capacitors. *Phys. Chem. Chem. Phys.* **15,** 8692-8699 (2013).
