# Supplementary material for: A supramolecular gel-elastomer system for soft iontronic adhesives
Source: Nat Commun. 2023 Apr 8;14:1990. doi: 10.1038/s41467-023-37535-4 (PMC10082814; doi:10.1038/s41467-023-37535-4)
Supplement: Supplementary file 3 — Description to Additional Supplementary Information [file 41467_2023_37535_MOESM3_ESM.pdf]

Description of Additional Supplementary Files

Supplementary Video 1 | OHGel-SHPU bilayer under cyclic tensile test.

Supplementary Video 2 | Metallic cube handling and weightlifting of the iontronic gripper.

Supplementary Video 3 | Heat/freeze tolerance of the OHGel-SHPU material system.

Supplementary Video 4 | Iontronic gripper picking up an icing/scorching metallic cube.

Supplementary Video 5 | Iontronic gripper gently handling delicate objects.

Supplementary Video 6 | Iontronic gripper picking up flat/tiny objects.

Supplementary Video 7 | Two-fingered iontronic gripper driven by < 400 V voltage input.

Supplementary Video 8 | Exteroceptive sensing and real-time display of the iontronic gripper.
