# Supplementary material for: A supramolecular gel-elastomer system for soft iontronic adhesives
Source: Nat Commun. 2023 Apr 8;14:1990. doi: 10.1038/s41467-023-37535-4 (PMC10082814; doi:10.1038/s41467-023-37535-4)
Supplement: Supplementary file 6 — Lasing Reporting Summary [file 41467_2023_37535_MOESM6_ESM.pdf]

nature research

Corresponding author(s): Pooi See Lee

Lasing Reporting Summary

Nature Research wishes to improve the reproducibility of the work that we publish. This form is intended for publication with all accepted papers reporting claims of lasing and provides structure for consistency and transparency in reporting. Some list items might not apply to an individual manuscript, but all fields must be completed for clarity.

For further information on Nature Research policies, including our data availability policy, see Authors & Referees.

Experimental design

Please check: are the following details reported in the manuscript?

1. Threshold

Item	Yes	No	Explanation
Plots of device output power versus pump power over a wide range of values indicating a clear threshold	☐	☒	The laser in Raman Microscope (Alpha300 R) is a commercial product. Its threshold information doesn't pertain to our test and can be obtained from the manufacturer (WiTec) upon reasonable request.

2. Linewidth narrowing

Item	Yes	No	Explanation
Plots of spectral power density for the emission at pump powers below, around, and above the lasing threshold, indicating a clear linewidth narrowing at threshold	☐	☒	The laser in Raman Microscope (Alpha300 R) is a commercial product. Its linewidth narrowing information doesn't pertain to our test and can be obtained from the manufacturer (WiTec) upon reasonable request.
Resolution of the spectrometer used to make spectral measurements	☒	☐	Supplementary Information, Supplementary methods, page 9.

3. Coherent emission

Item	Yes	No	Explanation
Measurements of the coherence and/or polarization of the emission	☐	☒	Irrelevant to our experiment.

4. Beam spatial profile

Item	Yes	No	Explanation
Image and/or measurement of the spatial shape and profile of the emission, showing a well-defined beam above threshold	☐	☒	Irrelevant to our experiment.

5. Operating conditions

Item	Yes	No	Explanation
Description of the laser and pumping conditions<br>*Continuous-wave, pulsed, temperature of operation*	☒	☐	Supplementary Information, Supplementary methods, page 9.
Threshold values provided as density values (e.g. W cm^{-2} or J cm^{-2}) taking into account the area of the device	☐	☒	The laser in Raman Microscope (Alpha300 R) is a commercial product. It provides a 35 mW power output at 633 nm, yet the power density is unknown and not necessarily needed to perform the experiment.

6. Alternative explanations

Item	Yes	No	Explanation
Reasoning as to why alternative explanations have been ruled out as responsible for the emission characteristics<br>*e.g. amplified spontaneous, directional scattering; modification of fluorescence spectrum by the cavity*	☐	☒	Irrelevant to our experiment.

7. Theoretical analysis

Item	Yes	No	Explanation
Theoretical analysis that ensures that the experimental values measured are realistic and reasonable<br>*e.g. laser threshold, linewidth, cavity gain-loss, efficiency*	☐	☒	Irrelevant to our experiment.

8. Statistics

Item	Yes	No	Explanation
Number of devices fabricated and tested	☐	☒	Irrelevant to our experiment.
Statistical analysis of the device performance and lifetime (time to failure)	☐	☒	Irrelevant to our experiment.
